# Targeting Hyperglycemic Bone Pre‐Metastatic Niche for Breast Cancer Bone Metastasis Therapy

**DOI:** 10.1002/advs.202504924

**Published:** 2025-06-10

**Authors:** Jianxin Ye, Peng Hu, Rui Zhang, Lei Zhou, Zonghua Luo, Yanan Chen, Shengzhe Ruan, Mengyi Zhu, Huaze Ding, Yike Qian, Yan Xing, Tong Meng, Changping Wang, Dianwen Song

**Affiliations:** ^1^ Department of Orthopedics Shanghai General Hospital Shanghai Jiao Tong University School of Medicine Shanghai 200080 China; ^2^ Department of Nuclear Medicine Shanghai General Hospital Shanghai Jiao Tong University School of Medicine Shanghai 200080 China; ^3^ School of Biomedical Engineering ShanghaiTech University Shanghai 200080 China; ^4^ School of Health Science and Engineering University of Shanghai for Science and Technology Shanghai 200080 China

**Keywords:** bone pre‐metastatic niches, engineered enzyme, glucose metabolism, selective chemotherapy, self‐amplifying, starvation

## Abstract

Bone is the most common site of breast cancer metastasis, yet understanding the intricate mechanisms and potential therapeutic targets remains nascent. Here it is reported that breast cancer establishes a hyperglycemic bone pre‐metastatic niche before migrating to bone tissue and further enhances glucose metabolism following metastatic colonization. An intervention strategy is subsequently proposed targeting glucose metabolism utilizing a biomimetic‐engineered enzyme‐based nanoplatform. This platform's membrane shielding reduces the interaction between engineered glucose oxidase and circulating glucose, while the engineered enzyme specifically targets glucose metabolism, enabling self‐amplifying starvation combined with selective chemotherapy. Such precision can precisely inhibit breast cancer bone metastases and block distal tumor dissemination. This study provides novel insights into the role of glucose metabolism in the pre‐metastatic niche and presents a proof‐of‐concept for metabolic‐targeted strategies in breast cancer bone metastasis treatment. This approach holds significant promise for improving therapeutic outcomes in metastatic breast cancer by targeting the metabolic vulnerabilities of the bone microenvironment and halting systemic tumor spread.

## Graphical abstract

This study reveals that breast cancer creates a hyperglycemic pre‐metastatic niche in bone prior to colonization and amplifies glucose metabolism post‐metastasis. To target this mechanism, a biomimetic enzyme‐engineered nanoplatform that disrupts tumor glycolytic pathways, proposing a therapeutic strategy to inhibit breast cancer bone metastasis by metabolic modulation is developed.

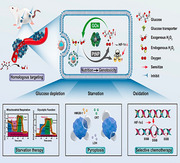

## 1 Introduction

Breast cancer has become the most prevalent malignancy worldwide, surpassing lung cancer as the leading tumor type among women.^[^ ^1^, ^2^ ^]^ Despite significant advancements in early detection and treatment, metastatic progression remains the principal cause of mortality, with bone metastasis being one of the most frequent and debilitating complications.^[^ ^3^, ^4^, ^5^ ^]^ In patients with advanced breast cancer, over 70% experience bone metastasis, which often leads to skeletal‐related adverse events such as fractures, severe pain, and loss of mobility. These events not only significantly impair the quality of life but also contribute to accelerated mortality.^[^ ^6^ ^]^ Recent studies have highlighted the complex and dynamic interactions between breast cancer cells and the bone microenvironment, underscoring the pivotal role of the bone in the progression of metastasis. Furthermore, reprogramming of both tumor cells and the bone niche facilitates the dissemination of metastases to distant organs, including the lungs, liver, and brain.^[^ ^7^, ^8^ ^]^ Thus, understanding the mechanisms driving bone metastasis is essential for developing effective strategies to prevent systemic tumor spread and improve clinical outcomes. Despite recent advances, our understanding of the intricate molecular and metabolic alterations associated with bone metastasis in breast cancer remains limited, and therapeutic approaches to target this devastating complication are still in their infancy.

Traditionally regarded as a passive structural component, the skeletal system is now recognized as a highly active organ with endocrine functions that significantly influence systemic metabolism. Bone tissue, particularly within the marrow, plays an integral role in regulating glucose metabolism, exhibiting a distinct capacity for glucose uptake.^[^ ^9^, ^10^, ^11^ ^]^ This function has been further highlighted by clinical studies showing that bone tissues harbor a high concentration of glucose, a key metabolic substrate.^[^ ^12^ ^]^ Epidemiological studies have also revealed an association between breast cancer and systemic disruptions in glucose homeostasis, further suggesting that metabolic alterations may contribute to the disease's progression.^[^ ^13^, ^14^, ^15^ ^]^ Moreover, emerging research has demonstrated that breast cancer cells can perturb systemic glucose regulation through exosome‐mediated disruption of pancreatic insulin secretion, further complicating the metabolic landscape in cancer patients.^[^ ^16^ ^]^ The target organs of cancer metastasis are not merely passive recipients of disseminated tumor cells but are actively and selectively remodeled by the primary tumor before the dissemination of cancer cells to the initial site.^[^ ^17^ ^]^ This dysregulated glucose metabolism has important implications for tumor biology, particularly in the context of bone metastasis, where changes in the local metabolic microenvironment may enhance tumor cell survival, proliferation, and colonization.

In addition to its role as a metabolic regulator, the bone microenvironment actively participates in the establishment of the pre‐metastatic niche—a concept that has gained significant attention in cancer research.^[^ ^18^, ^19^, ^20^ ^]^ The pre‐metastatic niche refers to the alterations in target organs, such as the bone, that precede and facilitate the colonization of disseminated tumor cells. These changes are orchestrated by the primary tumor through the release of soluble factors, extracellular vesicles, and extracellular matrix remodeling. While much has been learned about the cellular and molecular components of pre‐metastatic niches in other organs, the specific metabolic conditions that contribute to the formation of the bone pre‐metastatic niche remain poorly understood.^[^ ^21^, ^22^, ^23^, ^24^ ^]^ Given the intricate interplay among tumor dynamics, glucose metabolism, and the bone microenvironment, we hypothesize that breast cancer exerts distal regulatory effects on glucose distribution within bone tissues, and glucose metabolism emerges as a pivotal therapeutic target in addressing bone metastasis associated with breast cancer.

Targeting cancer metabolism represents an attractive strategy for therapeutic intervention, yet it presents significant challenges. One of the primary hurdles is achieving selective antitumor efficacy while minimizing toxicity to normal tissues. The metabolic pathways targeted by anticancer therapies are often shared by both tumor and normal cells, making it difficult to selectively target tumor cells without affecting healthy tissues.^[^ ^25^ ^]^ This is particularly problematic in the bone microenvironment, where tumor cells interact with a complex mixture of normal cells, encompassing various immune cells and stromal cells,^[^ ^26^ ^]^ along with various bone‐specific cells in the bone microenvironment like osteocytes, osteoblasts, and osteoclasts.^[^ ^27^ ^]^ Considering the overlap in many metabolic pathways crucial for the survival of both tumor and normal cells, particularly glucose metabolism, the therapeutic index for metabolic drugs tends to be restricted, potentially inducing considerable toxicity. In essence, metabolic‐targeted therapies, distinct from those aimed at genomic alterations, confront the significant hurdle of minimizing adverse impacts on normal cellular functions. Drug specificity remains a paramount concern in metabolic targeting.^[^ ^28^, ^29^ ^]^ Moreover, the ability of tumor cells to alter metabolic pathways introduces drug resistance, reducing the effectiveness of targeted treatments.^[^ ^30^, ^31^, ^32^, ^33^ ^]^ Consequently, approaches that incorporate combination therapies or block multiple pathways might present superior benefits compared to single‐agent therapies.

**Figure 1:**
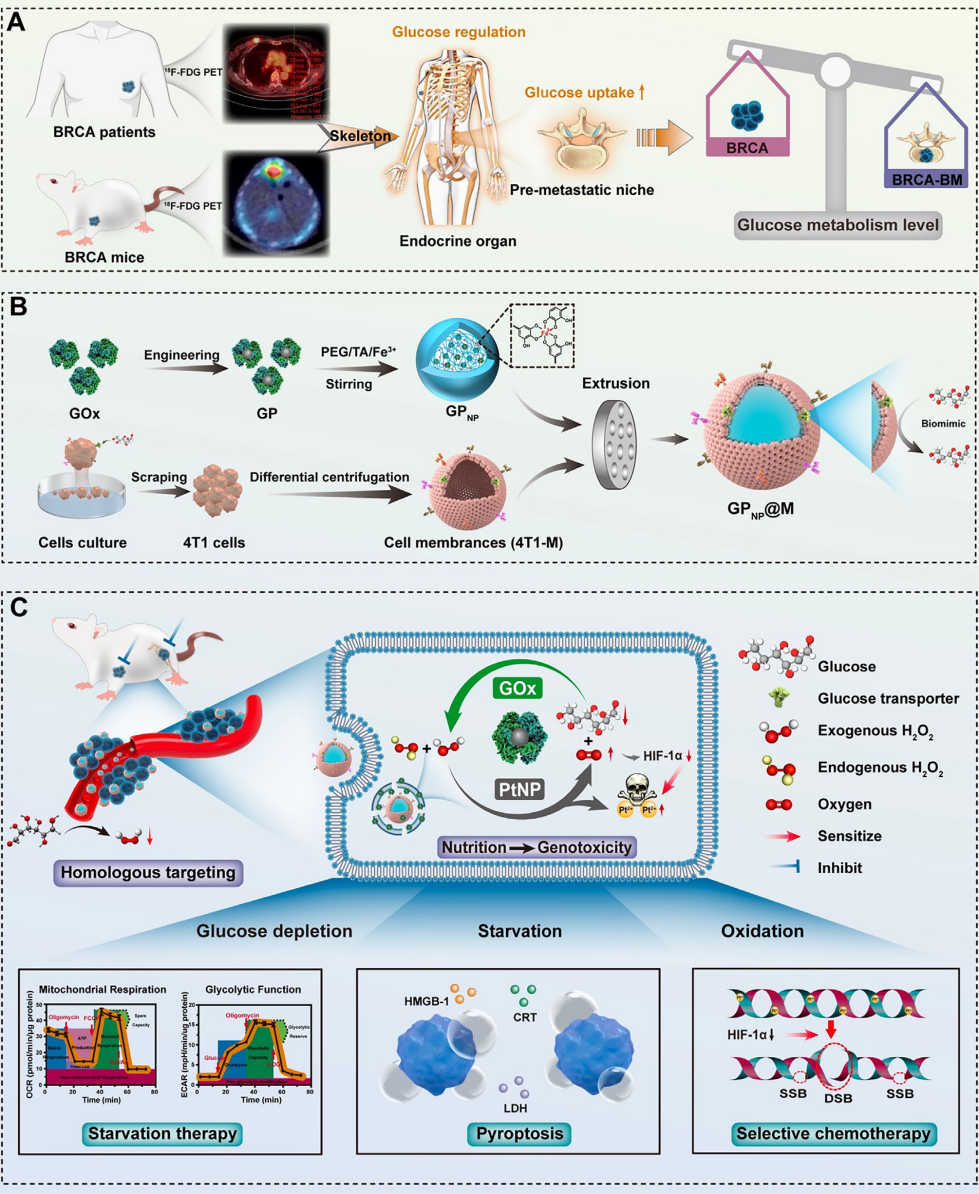
A novel therapeutic modality for breast cancer bone metastasis based on biomimetic and glucose metabolism‐targeted nanomedicines. A) Schematic representation illustrating glucose metabolism as a potential target for treating bone metastasis in breast cancer. B) Schematic diagram of the designing of a biomimetic nanomedicine targeting glucose metabolism. C) Schematic illustration of GP_NP_@M‐mediated homologous targeting and GP_NP_‐inducted self‐amplified starvation in coordination with selective chemotherapy for breast cancer bone metastasis. BRCA: orthotopic breast cancer; BRCA‐BM: breast cancer bone metastasis.

In this study, we discovered that breast cancer establishes a hyperglycemic bone pre‐metastatic niche before migrating to bone tissue and further enhances glucose metabolism following metastatic colonization. Based on the complex interplay between tumor dynamics and the bone hyperglycemic microenvironment, we further constructed a biomimetic‐engineered enzyme‐based nanoplatform (GP_NP_@M). This platform's membrane shielding reduces the interaction between engineered glucose oxidase (GP) and circulating glucose. Once internalized by breast cancer cells, GP is directed to attenuate intracellular glucose levels and induce starvation therapy effects. Additionally, platinum nanoparticles (PtNP) encapsulated within glucose oxidase (GO_X_) not only enhance the catalytic activity of GO_X_ but also facilitate a synergistic effect for selective chemotherapy within the tumor microenvironment. In conclusion, based on the hyperglycemic bone pre‐metastatic niche induced by breast cancer, we proposed a biomimetic‐engineered enzyme‐based nanoplatform with the capability of self‐amplifying starvation combined with selective chemotherapy. This platform holds promise for precisely mitigating breast cancer bone metastases and halting distal tumor dissemination (**Figure** **1**). This approach represents a significant step forward in the development of therapies that target the metabolic vulnerabilities of metastatic breast cancer, with the potential to improve patient outcomes and extend survival.

## 2 Results and Discussion

### 2.1 Hyperglycemic Bone Pre‐Metastatic Niche Induced by Breast Cancer

**Figure 2:**
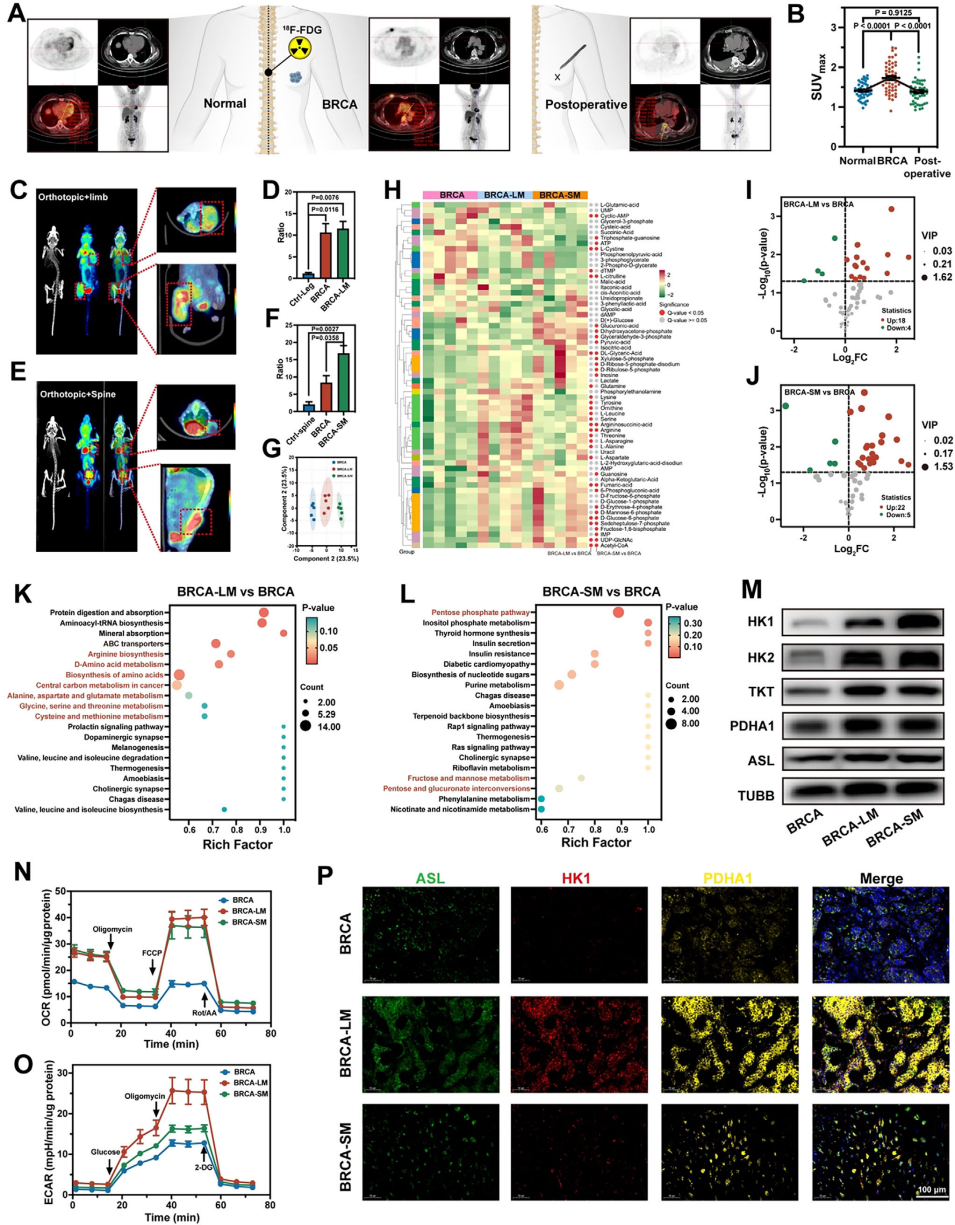
Glucose metabolism analysis of breast cancer bone metastasis. A,B) The ^18^F‐FDG PET‐CT images (A) and ^18^F‐FDG uptake quantification (B) of the spine in healthy individuals, breast cancer patients, and postoperative breast cancer patients (*n* = 50 independent samples, mean ± s.e.m.). C,D) PET/CT images (C) and ^18^F‐FDG uptake quantification (D) of BRCA and BRCA‐LM in the same mouse (*n* = 3 independent samples, mean ± s.e.m.). E,F) PET/CT images (E) and ^18^F‐FDG uptake quantification (F) of BRCA and BRCA‐SM in the same mouse (*n* = 3 independent samples, mean ± s.e.m.). G) OPLS‐DA of metabolites in three groups (*n *= 5 independent samples). H–L) Heatmap (H), Volcano map (I,J), and KEGG pathway enrichment analysis (K,L) of energy metabolism‐related metabolites in BRCA, BRCA‐LM, and BRCA‐SM (*n* = 5 independent samples). M) Western blotting analysis of corresponding key metabolic enzymes. N,O) Seahorse detection of primary tumor cells from BRCA, BRCA‐LM, and BRCA‐SM (*n* = 3 independent samples, mean ± s.e.m.). P) The analysis of key metabolic enzyme expression in human tumor samples. Ratio = SUV_ROI_/SUV_muscle_. *P*‐values were calculated by one‐way ANOVA with Tukey's multiple‐comparisons test (B) and two‐tailed unpaired Student *t*‐test (D,F). In M and P, representative results were displayed from at least triplicate independent experiments.

To investigate the association between bone metastasis and glucose metabolism in breast cancer, we employed positron emission tomography/computed tomography (PET/CT) imaging to assess glucose uptake at the common site of bone metastasis in breast cancer. We compared the uptake of fludeoxyglucose (^18^F‐FDG) in the spine (**Figure** **2**A,B), pelvis (Figure S1A,D, Supporting Information), rib (Figure S1B,E, Supporting Information), and femur (Figure S1C,F, Supporting Information) of healthy individuals, orthotopic breast cancer (BRCA) patients, and postoperative breast cancer patients. In BRCA patients, ^18^F‐FDG uptake was significantly elevated in these bone sites compared to healthy controls, with levels returning to normal postoperatively, suggesting that primary breast tumors may induce high metabolic activity in bone pre‐metastatic niches. Subsequently, we examined glucose metabolism in BRCA, limb metastasis (BRCA‐LM), and spinal metastasis (BRCA‐SM) mouse models using PET/CT scanning. Consistent with previous studies highlighting the role of the skeleton in glucose homeostasis, a prominent ^18^F‐FDG signal was detected in the spines of healthy mice (Figure S1G, Supporting Information). Notably, axial PET/CT scans showed elevated glucose uptake in the right forelimb (tumor side) (Figure S1H,I, Supporting Information) and spine (Figure S1J,K, Supporting Information) of mice with mammary carcinoma, indicating local metabolic alterations in response to breast cancer. We subsequently developed a 4T1 mammary cancer and metastasis model in the same mouse to replicate the metastatic process of clinical breast cancer and monitored glucose uptake at tumor sites (Figure 2C–F; Figure S1L–O, Supporting Information). Compared to BRCA, both BRCA‐LM, and BRCA‐SM showed higher ^18^F‐FDG uptake, likely influenced by the breast cancer‐induced metabolic upregulation in bone pre‐metastatic niches and the bone's glucose‐rich microenvironment.

Tumor samples from the three sites underwent targeted energy metabolomics analysis. Orthogonal partial least squares‐discriminant analysis (OPLS‐DA) analysis unveiled distinct metabolic phenotypes across the groups (Figure 2G). Integrating the heatmap (Figure 2H) and volcano plots (Figure 2I,J), numerous metabolites, including amino acids, nucleotides, organic acids, and phosphate sugars, were found to be upregulated in both BRCA‐LM and BRCA‐SM compared to the control group. Kyoto Encyclopedia of Genes and Genomes (KEGG) pathway enrichment analysis revealed that upon breast cancer cell colonization from the mammary fat pad to the bone (limbs and spine), the differentially expressed metabolites were enriched in amino acid metabolism pathways (highlighted in red), intricately associated with the tricarboxylic acid cycle (TCA) cycle (Figure 2K), and carbohydrate metabolism pathways (also highlighted in red, particularly emphasizing the pentose phosphate pathway) (Figure 2L), respectively, indicating activation of energy metabolism. Additional heatmaps (Figure S2A,B, Supporting Information) and Venn diagrams (Figure S2C, Supporting Information) displayed the differential metabolites between the comparative groups. We highlighted the metabolites (8/11) directly involved in energy metabolism among the shared differential metabolites using violin plots in Figure S2D (Supporting Information), encompassing pathways such as glycolysis (glucose‐6‐phosphate and acetyl‐CoA), pentose phosphate pathway (sedoheptulose‐7‐ phosphate and glyceric acid), and arginine biosynthesis (L‐citrulline, argininosuccinate acid, fumaric acid, and arginine) (Figure S2E, Supporting Information). To delve deeper into the mechanisms by which the intensity of metabolites changes between groups. The enzyme expressions upstream of correlated metabolites (Figure S2E, Supporting Information) were checked. As illustrated in Figure 2M and Figure S3A–E (Supporting Information), the key metabolic enzymes including hexokinase (HK1/2), transketolase (TKT), pyruvate dehydrogenase (PDHA1), and argininosuccinate lyase (ASL) were upregulated dramatically, further demonstrating vigorous glucose metabolism in both BRCA‐LM and BRCA‐SM. Moreover, primary tumor cells were isolated from the three tumor groups for cell metabolic analysis (Figure 2N,O; Figure S3F–L, Supporting Information), revealing significantly elevated oxygen consumption rate (OCR) and extracellular acidification rate (ECAR) levels in both BRCA‐LM and BRCA‐SM compared to BRCA. To explore whether this enhanced glucose metabolism exists in human bone metastases, we analyzed the expression levels of key metabolic enzymes (ASL, HK1, and PDHA1) in human tumor samples. Immunofluorescence analysis revealed elevated expression levels of these enzymes in bone metastases compared to BRCA tissues (Figure 2P), thus confirming the upregulation of glucose metabolism during bone metastasis in humans.

In summary, breast cancer establishes a hyperglycemic bone pre‐metastatic niche prior to metastasis to bone tissue and further enhances glucose metabolism following metastatic colonization. The complex interplay between tumor dynamics and the bone hyperglycemic microenvironment suggests that targeting glucose deprivation in bone metastases of breast cancer could be an effective therapeutic strategy.

### 2.2 Design and Characterization of GP_NP_@M

**Figure 3:**
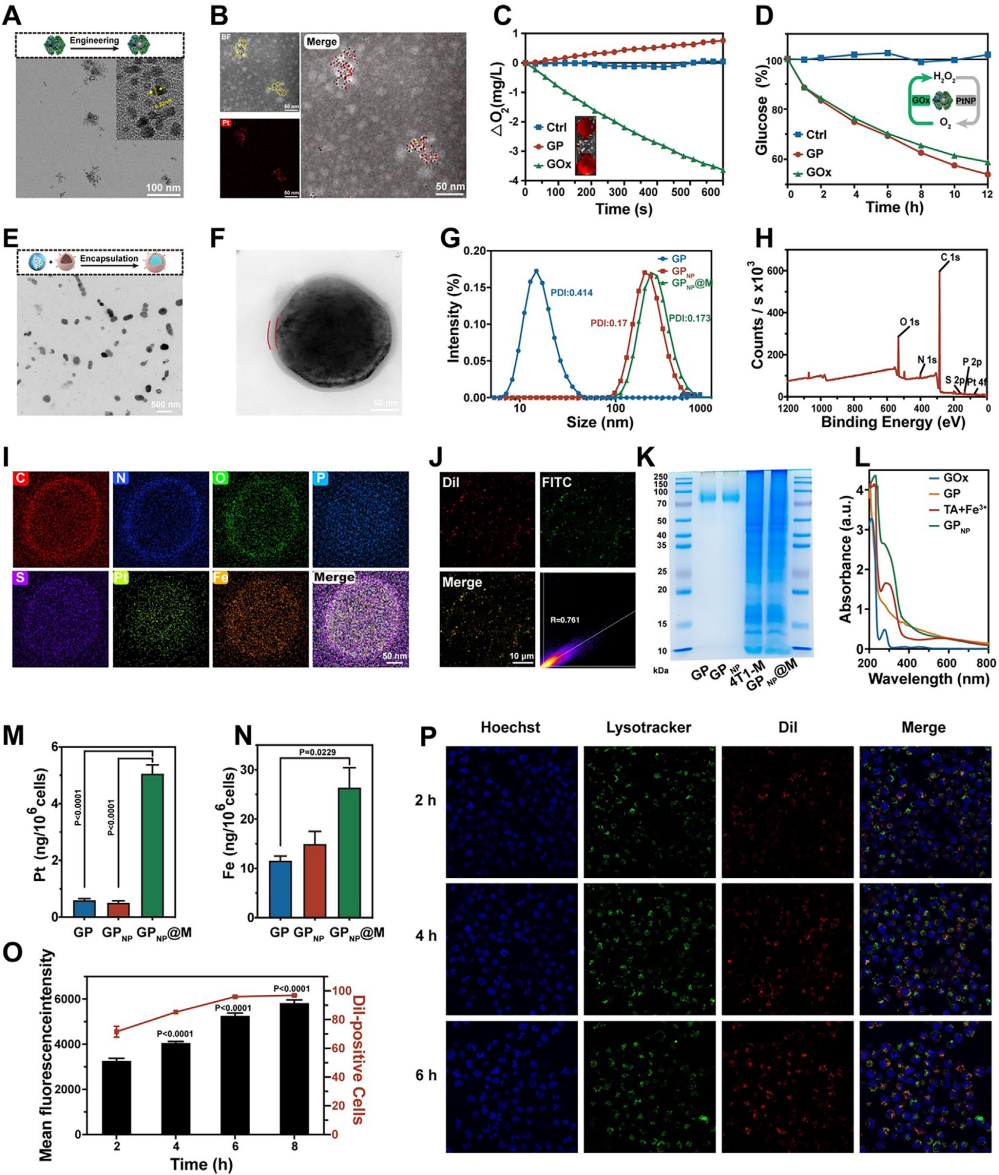
Synthesis, Characterization, and Cellular Internalization of GP_NP_@M. A) High‐resolution transmission electron microscopy (HR‐TEM) image of GP with an inset showing a lattice fringe of 0.22 nm. B) High‐angle annular dark‐field scanning transmission electron microscopy image and elemental mapping of negatively stained GP. C) Oxygen generation from GP and GOx in glucose and H_2_O_2_ mixed solution, with the inset depicting IVIS images of an oxygen‐sensitive probe under various conditions. D) Glucose consumption by GP and GOx in a glucose solution. E, F) TEM image (E) and HR‐TEM image (F) of GP_NP_@M. G) Hydrodynamic size distribution of GP, GP_NP_, and GP_NP_@M. H–J) XPS wide‐scan spectrum (H), EDS elemental mapping (I), and confocal fluorescent images (J) of GP_NP_@M (Red: Dil‐labeled 4T1‐M; Green: FITC‐labeled GP_NP_; Yellow: merge showing GP_NP_@M). K) Sodium dodecyl sulfate‐polyacrylamide gel electrophoresis (SDS‐PAGE) analysis of GP, GP_NP_, 4T1‐M, and GP_NP_@M. L) UV−vis spectra of GOx, GP, TA+Fe^3+^, and GP_NP_. M,N) Intracellular content of Pt (M) and Fe (N) in 4T1 cells after treatment with GP, GP_NP_, and GP_NP_@M, quantified by ICP‐MS (*n* = 3 independent samples, mean ± s.e.m.). O) Flow cytometric analysis of GP_NP_@M internalization in 4T1 cells (*n* = 3 independent samples, mean ± s.e.m.). P) Time‐dependent confocal fluorescent images of 4T1 cells incubated with GP_NP_@M (Green: lysotracker, marking endosomes; Red: Dil‐labeled GP_NP_@M). *P*‐values were calculated by one‐way ANOVA with Tukey's multiple‐comparisons test (M,N) and two‐way ANOVA with Šidák's multiple‐comparison test (O). In A, B, E, F, I–K, and P, representative results were displayed from at least triplicate independent experiments.

The ultimate goal of studying cancer metabolism is to improve cancer care. On the basis of previous studies on enzyme‐mediated metabolism regulation,^[^ ^34^, ^35^ ^]^ we synthesized the engineered GOx with PtNP inside, referred to as GP (**Figure** **3**A,B; Figure S4A,B, Supporting Information). Due to the incorporation of PtNP, GP demonstrated significant catalase‐like activity (Figure S4C, Supporting Information). The presence of PtNP considerably reduced the consumption of oxygen and the generation of H_2_O_2_ during catalysis (Figure S4D–F, Supporting Information), and even reversed the typical oxygen consumption associated with GOx (Figure 3C). This engineering strategy conferred self‐amplifying enzymatic activity on GP (Figure 3D; Figure S4G, Supporting Information). Our approach strategically synthesizes PtNP within the internal cavity of GOx, which not only preserves the enzyme's activity but also ensures a precise 1:1 stoichiometric ratio.^[^ ^35^ ^]^ Such meticulous engineering optimizes the benefits of the cascade reaction, thereby positioning GP as a promising candidate for a novel anti‐tumor drug targeting glucose metabolism.

To enhance the specificity and biocompatibility of GP, we employed a biomimetic approach to encapsulate GP nanoparticles within a cancer cell membrane. This configuration reduces the interaction between GP and circulating glucose, leveraging the membrane's shielding effect.^[^ ^36^ ^]^ Additionally, the homologous targeting inherent to this design directs GP's activity specifically toward tumor cells, thus achieving precise and targeted regulation. GP_NP_ and GP_NP_@M with uniform spherical nanostructures were confirmed by transmission electron microscope (TEM) (Figure S4H, Supporting Information; Figure 3E). The 4T1‐M was coated on the surface of GP_NP_ could also be clearly observed (Figure 3F,G; Figure S4I, Supporting Information). The chemical element composition of GP_NP_@M was analyzed by X‐ray photoelectron spectroscopy (XPS) and energy dispersive X‐ray spectroscopy (EDS), as depicted in the elemental diagrams (Figure 3H,I; Figure S4J–M, Supporting Information). Analysis confirmed the presence of C, N, O, P, S, Pt, and Fe within GP_NP_@M, and it was specifically verified that Pt was fully encapsulated within the interior of the membrane. Colocalization studies, as demonstrated in Figure 3J, showed that Dil‐labeled 4T1‐M colocalized well with FITC‐labeled GP (*R* = 0.761). Gel electrophoresis confirmed that the protein bands in GP_NP_ were identical to those in GP, and similar bands were observed between GP_NP_@M and 4T1‐M (Figure 3K), indicating successful membrane encapsulation. The UV–vis spectrum revealed a characteristic peak at ≈270 nm for GP_NP_, attributed to the fusion of the coordination peak of TA+Fe^3+^ and the protein peak of GP (Figure 3L), further confirming the successful preparation of GP_NP_@M. These comprehensive characterizations underscore the targeted design and functional integrity of the biomimetic encapsulation.

Moreover, GP_NP_@M demonstrated robust stability in H_2_O, FFM (FBS‐free medium), and FM (FBS‐containing medium) attributable to the protective effects of the encapsulating membrane. This was demonstrated by the stability of the particle size over three days, in contrast to the rapid disassembly of GP_NP_ (Figure S4N,O, Supporting Information), which established a solid foundation for its application in anti‐tumor therapies in vivo. The therapeutic efficacy of nanomedicines is heavily dependent on cellular uptake. Inductively coupled plasma mass spectrometry (ICP‐MS) analysis of intracellular Pt and Fe levels confirmed that GP_NP_@M facilitated enhanced access to 4T1 cells compared to GP and GP_NP_ (Figure 3M,N). Flow cytometry analyses revealed a time‐dependent uptake of GP_NP_@M by 4T1 cells (Figure 3O). Additionally, the intensity of the red fluorescence signal from GP_NP_@M increased within 2–6 h, and there was notable colocalization with lysotracker‐stained endosomes (Figure 3P), suggesting that GP_NP_@M was effectively internalized by 4T1 cells via endosome‐mediated endocytosis pathways. To evaluate the off‐target effects of GP_NP_@M, we also examined the internalization of GP_NP_@M in different non‐tumor cell lines (HUVEC, MC3T3‐E1, and RAW264.7). As clearly shown in Figure S5 (Supporting Information), GP_NP_@M was efficiently internalized by 4T1 cells, while almost no red fluorescence of GP_NP_@M was observed in other cell types, indicating that the tumor cell membrane composition enhanced the targeting specificity of GP_NP_@M, thereby reducing off‐target effects.

### 2.3 Enhanced Autocatalytic Starvation‐Induced Pyroptosis Effect of GP_NP_@M

**Figure 4:**
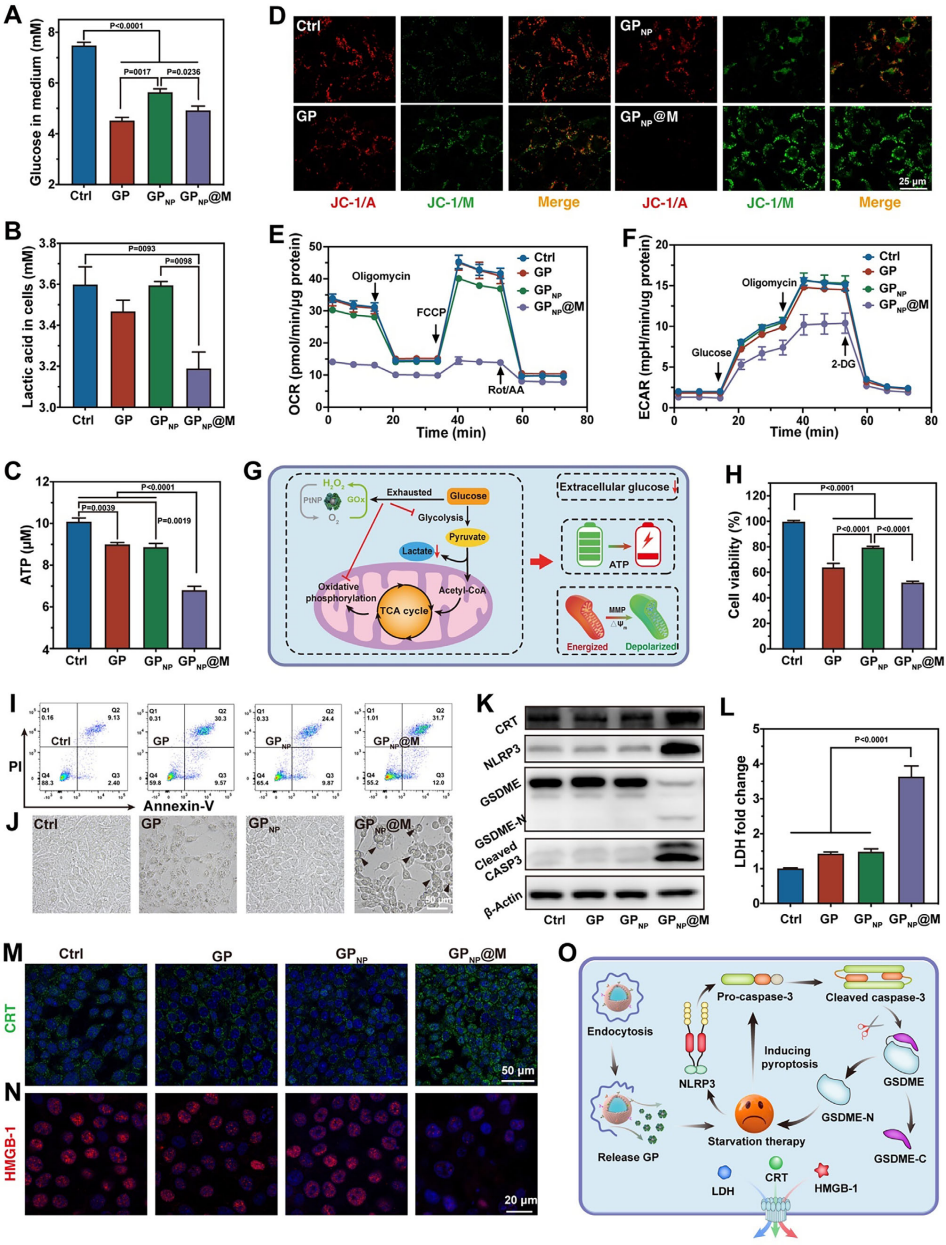
Starvation and pyroptosis induced by GP_NP_@M in vitro. A) Glucose concentration in the medium after treatment with PBS, GP, GP_NP_, and GP_NP_@M (*n* = 3 independent samples, mean ± s.e.m.). B,C) Levels of lactic acid (B) and ATP content (C) in 4T1 cells after different treatments (*n *= 3 independent samples, mean ± s.e.m.). D) JC‐1 staining of 4T1 after different treatments. E,F) The OCR (E) and ECAR (F) levels in 4T1 cells after different treatments for 24 h (*n* = 4 independent samples, mean ± s.e.m.). G) Schematic diagram illustrating the starvation effect exerted by GP_NP_@M. H,I) Viability (H) and flow cytometry (I) analysis of 4T1 cells treated with PBS, GP, GP_NP_, and GP_NP_@M (*n* = 5 independent samples, mean ± s.e.m.). J) The bright‐field morphology of 4T1 cells after various treatments, with a black arrow indicating typical pyroptosis cells characterized by bubble‐like protrusions. K–N) Western blotting bands of pyroptosis‐related proteins (K), the release of LDH (L), and representative immunofluorescence staining of CRT (M) and HMGB1 (N) in 4T1 cells treated with different materials (*n* = 4 independent samples, mean ± s.e.m.). O) Schematic illustration of GP_NP_@M inducing pyroptosis through starvation effect. *P*‐values were calculated by one‐way ANOVA with Tukey's multiple‐comparisons test (A–C, H, and L). In D, I, J, K, M, and N, representative results were displayed from at least triplicate independent experiments.

Subsequently, we assessed the capacity of GP_NP_@M to deplete glucose at the cellular level in order to investigate its glucose starvation effect. As demonstrated in **Figure** **4**A, 4T1 cells treated with GP_NP_@M exhibited a significant reduction in extracellular glucose levels. This observation suggested that GP_NP_@M was effectively internalized, thereby facilitating greater glucose uptake from the culture medium, matching the self‐amplifying glucose decomposition activity observed with GP (Figure 3D). In contrast, GP_NP_ achieved only moderate glucose reduction, attributed to constraints in enzyme activity due to its structural assembly. We next analyzed the impact of these treatments on the production of intracellular lactate and adenosine triphosphate (ATP), which are closely linked to intracellular glucose levels. GP_NP_@M markedly suppressed the generation of lactic acid and ATP within cells, while GP and GP_NP_, acting through extracellular catalysis, exerted a less pronounced effect on these metabolic processes (Figure 4B,C). Given the critical role of mitochondria in cellular health and metabolic function, we employed the JC‐1 dye to assess mitochondrial integrity (Figure 4D; Figure S6A, Supporting Information). GP_NP_@M induced more profound mitochondrial damage than GP or GP_NP_, as indicated by the loss of red fluorescence and the emergence of bright green fluorescence in the cytoplasm, signaling mitochondrial depolarization. Additionally, we quantified the OCR and ECAR rates in 4T1 cells following treatment. The group treated with GP_NP_@M consistently showed the lowest OCR values and a significant downturn in associated metabolic indicators (Figure 4E; Figure S6B–J, Supporting Information). Conversely, GP and GP_NP_ treatments caused negligible changes in OCR levels, reinforcing the evidence of severe mitochondrial dysfunction in cells treated with GP_NP_@M, aligning with the JC‐1 staining results. Moreover, 4T1 cells exposed to GP_NP_@M for both 12 and 24 h exhibited diminished responsiveness to ECAR inhibitors, as evidenced by the minimal glycolytic activity observed (Figure 4F; Figure S6K–Q, Supporting Information), indicative of inhibited glycolysis. Interestingly, GP displayed inconsistent effects on ECAR at 12 and 24 h (Figure 4F; Figure S6N, Supporting Information). This is probably because the initial reduction of extracellular glucose by GP facilitated increased glucose uptake by cells, which then adapted to the altered environmental conditions. Collectively, these results underscore the robust glucose deprivation effect induced by GP_NP_@M (Figure 4G), positioning it as a potent agent in anti‐tumor strategies targeting metabolic pathways.

The in vitro anti‐tumor efficacy of GP_NP_@M‐mediated starvation therapy was estimated, revealing that the tumor cell‐killing effects of various treatments closely aligned with glucose deprivation observed in Figure 4A (Figure 4H). This suggested that glucose deprivation played a critical role in inducing cell death. Supporting this observation, live/dead staining (Figure S7A, Supporting Information) and flow cytometric analysis (Figure 4I) results showed consistent effects on cell viability. Previous research has demonstrated that glucose depletion by GOx can trigger pyroptosis in tumor cells.^[^ ^37^, ^38^ ^]^ In line with these findings, 4T1 cells exposed to GP_NP_@M rapidly displayed hallmark features of pyroptosis, such as cellular enlargement and numerous bubble‐like protrusions (Figure 4J), distinguishing them from cells in other treatment groups. We also evaluated the expression of pyroptosis‐associated proteins. Significant downregulation of gasdermin E (GSDME) and upregulation of pyrin domain containing 3 (NLRP3), GSDME‐N terminal, and cleaved caspase‐3 (cleaved CASP3) were observed in the GP_NP_@M‐treated cells (Figure 4K; Figure S7B–E, Supporting Information), with this group also exhibiting the highest levels of lactate dehydrogenase (LDH) release (Figure 4L), a result of the distinctive pore‐forming activity of the GSDME‐N domain.^[^ ^39^, ^40^ ^]^ Additionally, GP_NP_@M treatment significantly increased calreticulin (CRT) expression (Figure 4K,M; Figure S7F, Supporting Information) and high‐mobility group box 1 (HMGB‐1) release (Figure 4N), which are indicative of enhanced immunogenicity due to pyroptosis. Overall, GP_NP_@M promoted the release of damage‐associated molecular patterns (DAMPs) through starvation‐induced pyroptosis,^[^ ^41^ ^]^ thereby enhancing its potential for effective tumor eradication (Figure 4O).

### 2.4 Tumor Microenvironment‐Responsive Selective Chemotherapy and Hypoxia Amelioration

**Figure 5:**
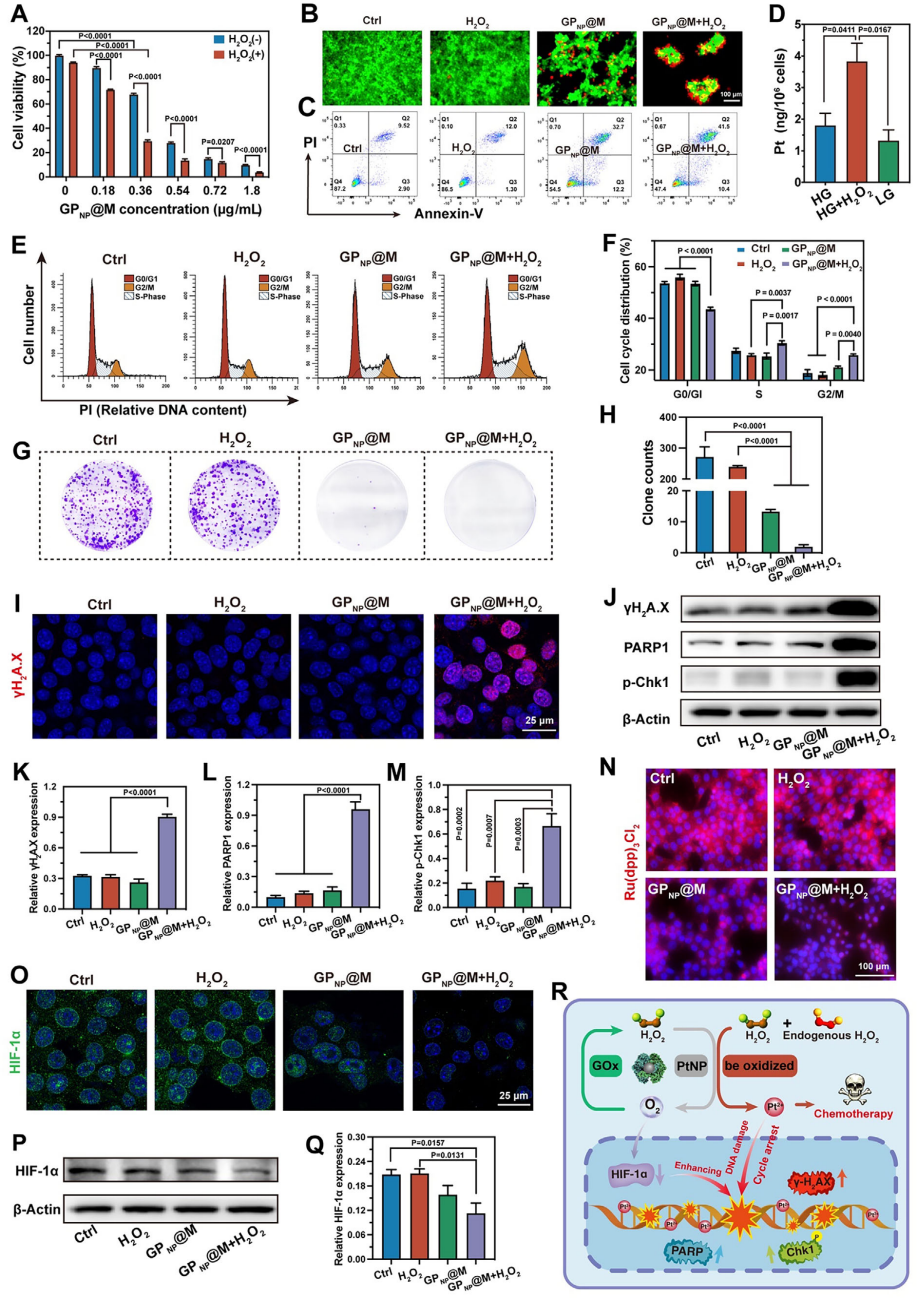
GP_NP_@M exerted a selective chemotherapy effect. A) Viability of 4T1 cells after treatment with different GOx concentrations of GP_NP_@M with or without H_2_O_2_ (*n* = 5 independent samples, mean ± s.e.m.). B,C) Live/dead staining (B) and corresponding flow cytometry analysis (C) after treatment with PBS, H_2_O_2_ (100 µM), GP_NP_@M, and GP_NP_@M+H_2_O_2_ at a GOx concentration of 0.36 µg mL^−1^. D) Pt content in the nucleus of 4T1 cells after incubating with GP_NP_@M in HG (glucose concentration: 4.5 g L^−1^) medium, HG medium with H_2_O_2_ (100 µm), and LG (glucose concentration: 1 g L^−1^) medium for 6 h (*n* = 3 independent samples, mean ± s.e.m.). E,F) Cell cycle analysis (E) and relative quantitative cycle distribution (F) in 4T1 cells after different treatments (*n* = 3 independent samples, mean ± s.e.m.). G,H) Cell colony formation assay (G) and relative quantitative colony numbers (H) in 4T1 cells treated with various formulations (*n* = 3 independent samples, mean ± s.e.m.). I,N,O) Representative fluorescence images of γH_2_A.X (I), Ru(dpp)_3_Cl_2_ (N), and HIF‐1α (O) in 4T1 cells after treatment with different materials. J–M,P,Q) Western blotting analysis of DNA damage markers (J) and HIF‐1α (P), along with relative quantitative protein expression levels (K–M,Q) in 4T1 cells with indicated treatment (*n* = 4 independent samples, mean ± s.e.m.). R) Schematic diagram of selective chemotherapy effect mediated by GP_NP_@M. *P*‐values were calculated by two‐way ANOVA with Šidák's multiple‐comparison test (A), two‐way ANOVA with Tukey's multiple‐comparisons test (F), and one‐way ANOVA with Tukey's multiple‐comparisons test (D,H,K–M,Q). In B, C, E, G, I, J, N, and O, representative results were displayed from at least triplicate independent experiments.

Short‐term fasting and fasting‐mimicking diets have been shown to sensitize tumor cells to chemotherapy, while concurrently shielding normal cells from stressor‐induced damage.^[^ ^42^ ^]^ Consequently, the integration of starvation therapy with adjunctive chemotherapy holds substantial therapeutic promise. Beyond its catalase‐like activity, ultra‐small PtNP exhibits selective chemotherapeutic properties in cellular environments with a high oxidation potential.^[^ ^43^ ^]^ H_2_O_2_ augmentation significantly elevated the cytotoxic response in 4T1 cells treated with GP_NP_@M, an effect attributable to the accelerated oxidation of Pt^0^ to cytotoxic Pt^II^, as evidenced by cell viability and apoptosis analysis (**Figure** **5**A–C). We then measured intranuclear platinum content, observing a twofold increase in Pt^II^ under high glucose (HG) conditions supplemented with H_2_O_2_ compared to glucose alone (Figure 5D), underscoring the role of Pt^II^ in DNA disruption.^[^ ^44^ ^]^ It is noteworthy that the platinum concentration in HG is slightly higher than that in low glucose (LG), suggesting that exogenous H_2_O_2_ produced by glucose oxidation can also contribute to oxidation processes. When exposed to combined glucose and H_2_O_2_ milieu, the proportion of Pt^II^ within the platinum nanoparticles surged from 22.7% to 68% (Figure S8A,B, Supporting Information), emphasizing the oxidative potential of this microenvironment to induce PtNP's cytotoxic state. Pt^II^‐induced DNA damage precipitated robust cell cycle arrest in 4T1 cells, as depicted in Figure 5E,F, where GP_NP_@M exposure, especially with concurrent H_2_O_2_, markedly impeded progression through the S and G2/M phases. This synergistic effect of nutrient deprivation and cell cycle inhibition markedly reduced 4T1 cell proliferation (Figure 5G,H). Further analysis of DNA damage response biomarkers revealed that the GP_NP_@M‐H_2_O_2_ combination precipitated the most substantial DNA damage, manifesting as elevated levels of phosphorylated histone H_2_AX (γH_2_A.X) detected through intensified immunofluorescence staining (Figure 5I) and immunoblot analyses (Figure 5J,K). This treatment paradigm also significantly upregulated other critical DNA damage markers, such as poly (ADP‐ribose) polymerase 1 (PARP1) and phosphorylated checkpoint kinase 1 (p‐CHK1), in the treated 4T1 cells (Figure 5J–M), reinforcing the potential of this combinatorial approach to induce significant genotoxic stress.

We subsequently explored the impact of GP_NP_@M on tumor oxygenation. The presence of exogenous H_2_O_2_ significantly quenched the red fluorescence of the oxygen probe in the GP_NP_@M‐treated cells (Figure 5N), indicating elevated oxygen levels. Hypoxia‐inducible factor 1 (HIF‐1α), a crucial regulator of cellular responses to oxygen fluctuations,^[^ ^45^ ^]^ typically enhances glucose transporter (GLUT) expression, which can undermine the efficacy of starvation therapies,^[^ ^46^, ^47^, ^48^ ^]^ and elevates multidrug resistance‐associated protein 1 (MRP1) levels, often leading to chemotherapy resistance.^[^ ^49^, ^50^ ^]^ Notably, the degradation of HIF‐1α was observed when GP_NP_@M treatment was combined with H_2_O_2_ (Figure 5O–Q), suggesting a decrease in these adaptive responses. These oxygen‐modulating effects are closely associated with the catalase‐like activity of PtNP, which improves hypoxia conditions, thereby enhancing the outcomes of both starvation therapy and chemotherapy. Importantly, GP_NP_@M induced a selective chemotherapeutic effect, optimized by the high oxidative potential of the tumor microenvironment. This was further supported by a reduction in HIF‐1α expression, which sensitizes tumors to chemotherapy (Figure 5R).

### 2.5 RNA‐Sequencing Analyses the Therapeutic Effect of GP_NP_@M

**Figure 6:**
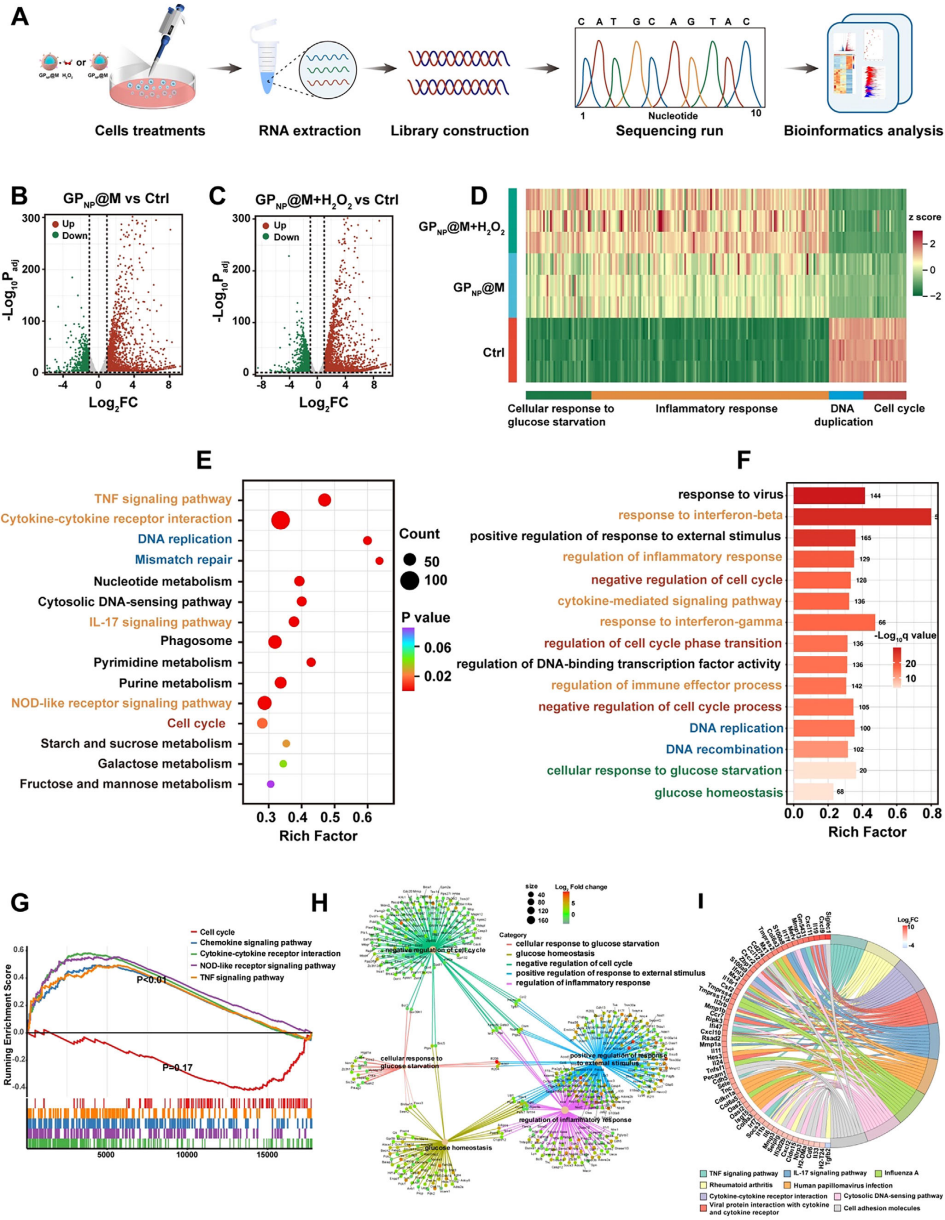
RNA‐sequencing analyses the therapeutic effect of GP_NP_@M. A) Schematic overview of the RNA‐seq experiment. B,C) Volcano plot of DEGs of cells treated with GP_NP_@M (B) or GP_NP_@M plus H_2_O_2_ (C). D) Heatmap of four types of gene expression levels (cellular response to glucose starvation, inflammatory response, DNA replication, and cell cycle) after treatments. E–H) KEGG pathway enrichment analysis (E), GO analysis (F), GSEA analysis (G), and gene‐concept network plot (H) of DEGs between the GP_NP_@M plus H_2_O_2_ treatment group and control group. I) The chord diagram of the KEGG pathway analysis of the common DEGs after the treatment with GP_NP_@M plus H_2_O_2_ versus GP_NP_@M. For each plot, *n* = 3 independent samples. In B and C, the boundary was |log_2_FC| > 1 and *P*_adj _< 0.05. *P*_adj_ was calculated by multiple hypothesis tests.

A transcriptomic analysis was conducted to further verify the internal mechanisms of GP_NP_@M‐induced starvation‐related pyroptosis and selective chemotherapy (**Figure** **6**A). A total of 3343 and 4978 differentially expressed genes (DEGs) were identified in GP_NP_@M and GP_NP_@M plus H_2_O_2_‐treated group, respectively, compared to the control group (Figure 6B,C). The heatmap of classified DEGs revealed that upregulated genes were predominantly involved in cellular response to glucose starvation and inflammatory pathways, whereas downregulated genes were associated with DNA replication and cell cycle regulation (Figure 6D). Obviously, GP_NP_@M plus H_2_O_2_‐treated group underwent more significant changes compared to GP_NP_@M treated group, due to the enhanced self‐amplifying enzyme activity and high oxidative potential‐induced chemotherapy of GP in the presence of H_2_O_2_.

KEGG pathway analysis showed significant enrichment in pathways related to inflammation (orange), DNA replication and repair (blue), and cell cycle (red) (Figure 6E; Figure S9A, Supporting Information). Besides, gene ontology (GO) enrichment analysis additionally validated the cellular response to glucose starvation response (green) (Figure 6F; Figure S9B, Supporting Information). Moreover, gene set enrichment analysis (GSEA) corroborated these findings, showing substantial activation of inflammation‐related pathways and suppression of cell cycle genes (Figure 6G; Figure S9C, Supporting Information). Gene‐concept network plots demonstrated a closer association between critical genes involved in the inflammatory response, glucose starvation regulation, and cell cycle arrest under the addition of H_2_O_2_ (Figure 6H; Figure S9D, Supporting Information). A Venn diagram (Figure S9E, Supporting Information) revealed 2978 common DEGs between the two comparison groups. To further explore the function roles of these genes, we performed a chord diagram of KEGG (Figure 6I), which showed the common DEGs were mainly enriched in the inflammation pathways. These results revealed that GP_NP_@M treatment promotes robust antitumor effects through enhancing autocatalytic starvation‐induced pyroptosis effect and DNA damage‐induced selective chemotherapy within the high‐oxidation tumor microenvironment.

### 2.6 In Vivo Therapeutic Efficacy and Mechanism against Breast Cancer

**Figure 7:**
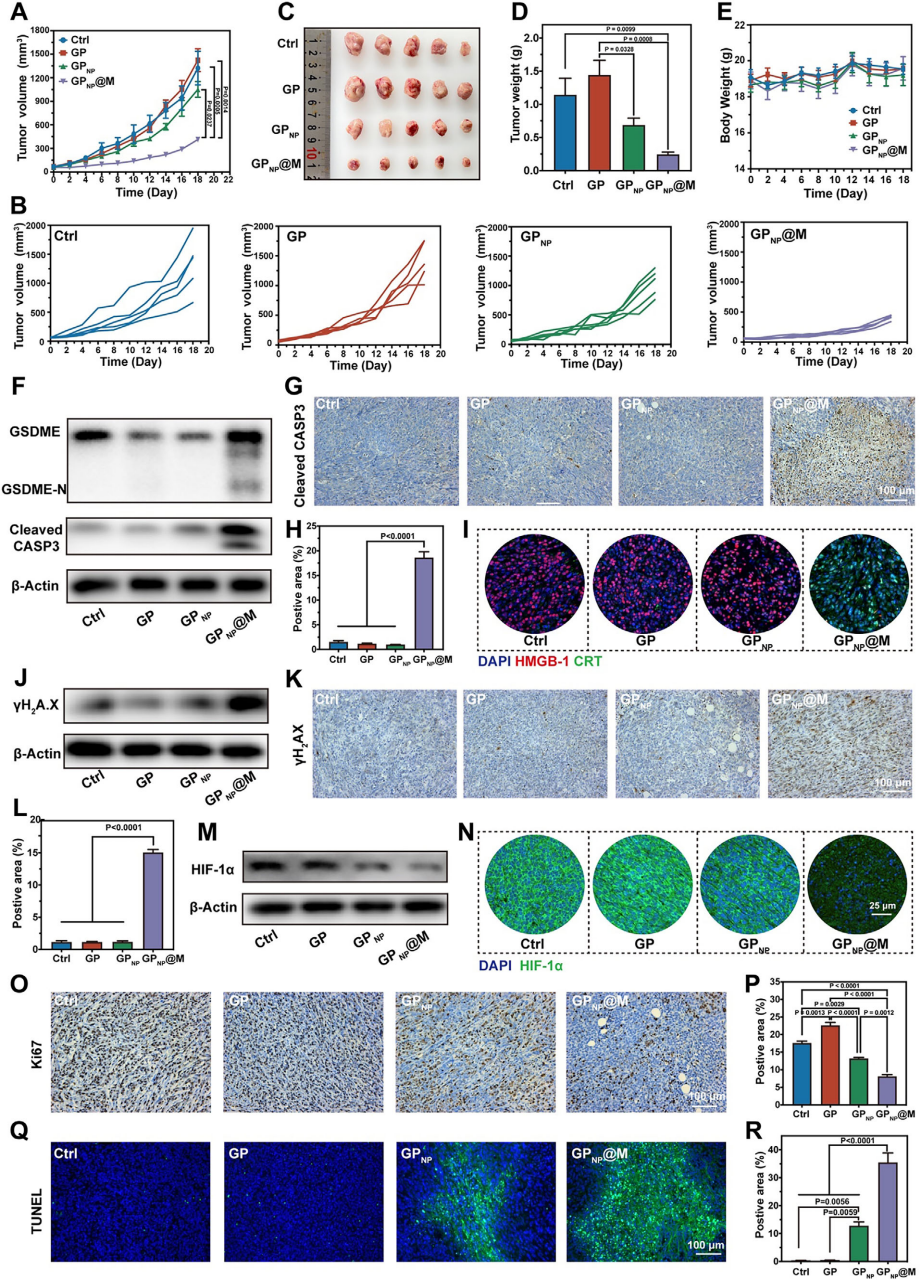
In vivo anti‐orthotopic breast cancer efficacy. A,B) Tumor growth curve (A) and individual tumor volume changes (B) in mice across four treatment groups (*n* = 5 independent samples, mean ± s.e.m.). C–E) Photograph of the extracted tumors (C), tumor weights (D), and corresponding changes in body weight (E) following various treatments (*n *= 5 independent samples, mean ± s.e.m.). F) Western blotting analysis of pyroptosis‐related protein expression in different treatment groups. G,H) Immunohistochemistry images (G) and quantitative data (H) of cleaved caspase‐3 in tumors from different groups (*n* = 3 independent samples, mean ± s.e.m.). I) Immunofluorescence images of HMGB‐1 (Red) and CRT (Green) in different treatment groups. J) Western blotting of DNA damage‐related protein expression in mice from different treatment groups. K,L) Immunohistochemistry images (K) and quantitative data (L) of γH_2_A.X in tumors from different groups (*n* = 3 independent samples, mean ± s.e.m.). M,N) Western blotting (M) and immunofluorescence staining (N) of HIF‐1α in tumor sections post‐treatment. O–R) Immunohistochemistry of Ki67 (O) and TUNEL staining (Q) in different groups, with relative quantitative analysis (P,R) (*n* = 3 independent samples, mean ± s.e.m.). *P*‐values were calculated by one‐way ANOVA with Tukey's multiple‐comparisons test (A,D,H, L,P,R). In F, G, I–K, M–O, and Q, representative results were displayed from at least triplicate independent experiments.

Leveraging the synergistic treatment mechanisms and targeted affinity,^[^ ^51^ ^]^ we then evaluated the antitumor efficacy of GP_NP_@M using an orthotopic 4T1‐tumor‐bearing mouse model. When tumors reached ≈50–80 mm^3^, the mice were stratified into four groups and received intravenous injections of PBS, GP, GP_NP_, or GP_NP_@M, respectively. As illustrated in **Figure** **7**A,B, the GP_NP_@M‐treated group exhibited the slowest tumor growth rate and the highest tumor inhibition rate of 68.8%, attributed to enhanced tumor targeting, efficient endocytosis, and the multifaceted tumoricidal properties of GP_NP_@M. The excised tumors were photographed and weighed (Figure 7C,D), further confirming the superior tumor suppression by GP_NP_@M. It is noteworthy that due to the enhanced permeability and retention effect, GP_NP_ also moderately reduced tumor growth. Additionally, there were no significant changes in the body weight of the tumor‐bearing mice across different treatments (Figure 7E), nor were there substantial differences in hematological indices (Figure S10A, Supporting Information) or histopathological examinations of major organs (heart, liver, spleen, lung, and kidney) in treated healthy mice (Figure S10B, Supporting Information). As displayed in Figure S11 (Supporting Information), GP_NP_@M demonstrated excellent hemocompatibility, showing no detectable hemolytic activity in mouse erythrocytes even at elevated GOx concentrations of 10 µg mL^−1^, indicating the favorable biocompatibility of GP_NP_@M.

To further elucidate the anti‐tumor mechanisms of GP_NP_@M in *vivo*, we investigated both pyroptosis‐related and genotoxicity‐related protein expressions in tumor tissues. Notably, elevated levels of cleaved caspase‐3 and the GSDME‐N domain were detected in tumors from mice treated with GP_NP_@M (Figure 7F–H; Figure S12A, Supporting Information). Additionally, enhanced exposure of CRT and release of HMGB‐1 were observed in the GP_NP_@M treatment group (Figure 7I), indicating that GP_NP_@M promotes pyroptosis within tumor tissues. In terms of selective chemotherapy, GP_NP_@M significantly increased the expression of γH_2_A.X, a critical marker of DNA damage (Figure 7J–L; Figure S12B, Supporting Information), and effectively downregulated HIF‐1α levels (Figure 7M,N; Figure S12C, Supporting Information) in tumors. Moreover, Ki‐67 staining (Figure 7O,P) and immunofluorescence terminal deoxynucleotidyl transferase‐mediated dUTP‐biotin nick end labeling (TUNEL) (Figure 7Q,R) showed the least cell proliferation and the most distribution of apoptotic cells in the GP_NP_@M group, further demonstrating the best therapeutic effect of GP_NP_@M among all treatments. These findings underscore that GP_NP_@M not only initiates pyroptosis but also sensitizes tumors to selective chemotherapy in vivo, thereby enhancing its therapeutic impact.

### 2.7 GP_NP_@M Inhibits Breast Cancer Bone Metastases and Distal Tumor Dissemination

**Figure 8:**
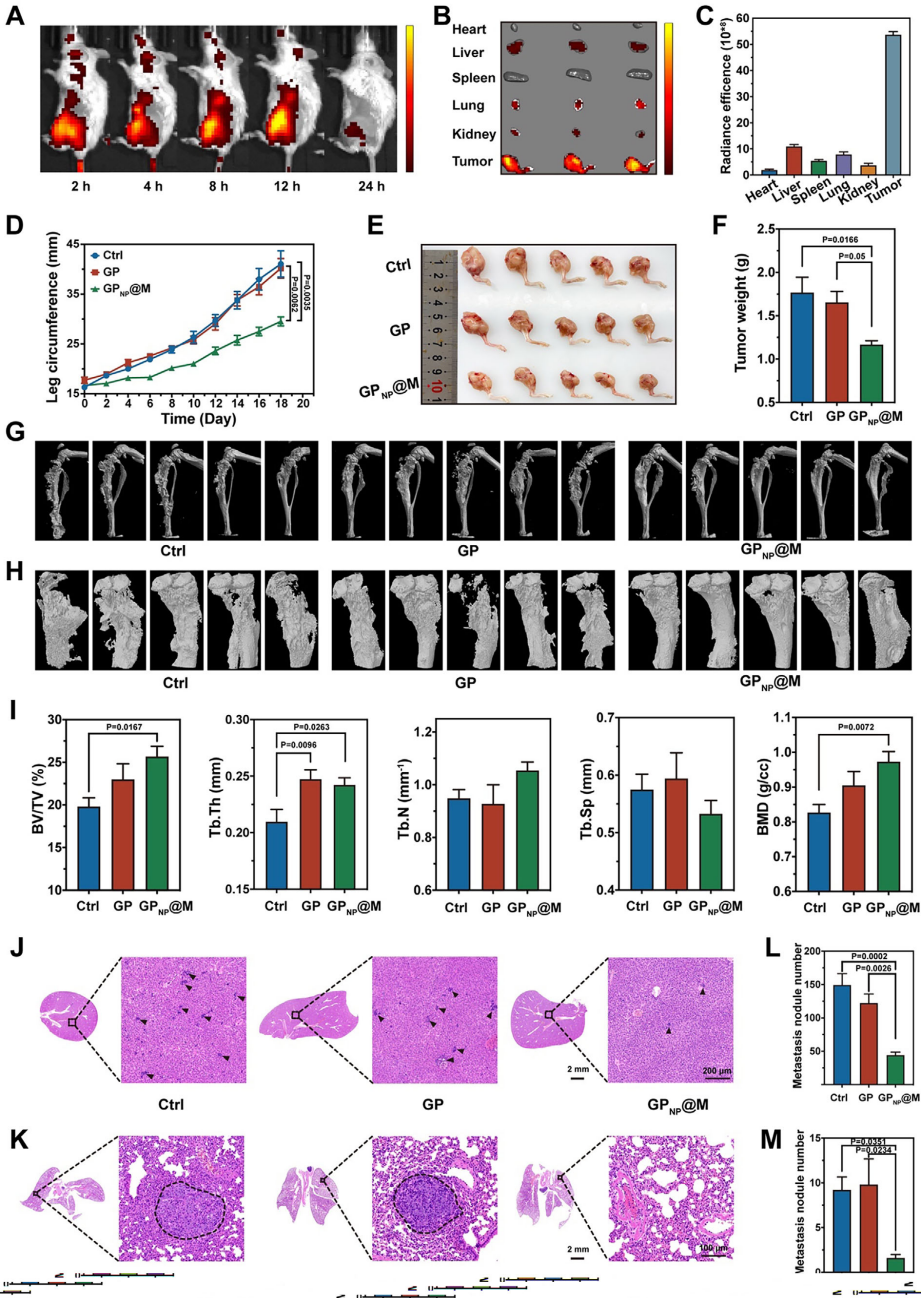
The therapeutic effect of GP_NP_@M on breast cancer bone metastasis. A) IVIS images of mice after administration of Cy5‐labeled GP_NP_@M at different time points. B,C) Fluorescence images (B) and quantitative analysis (C) of major organs at 24 h after administration (*n* = 3 independent samples, mean ± s.e.m.). D) Changes in leg circumference among mice after different administrations (*n* = 5 independent samples, mean ± s.e.m.). E,F) Gross photograph (E) and corresponding weight (F) of tumor‐bearing legs from each group (*n *= 5 independent samples, mean ± s.e.m.). G–I) Micro‐CT images (G), 3D reconstruction (H), and bone parameters (I) of tumor‐bearing tibias post‐treatments (*n* = 5 independent samples, mean ± s.e.m.). J,K) HE staining of the liver (J) and the lung (K) in each group. L,M) Statistical analysis of metastatic lesions in livers (L) and lungs (M) (*n *= 5 independent samples, mean ± s.e.m.). *P*‐values were calculated by one‐way ANOVA with Tukey's multiple‐comparisons test (D,F,I,L,M). In A, J, and K, representative results were displayed from at least triplicate independent experiments.

Upon identifying glucose metabolism as a promising therapeutic target for the treatment of breast cancer bone metastasis and substantiating the therapeutic potency of GP_NP_@M in orthotopic breast tumors, we expanded our research to examine the impact of this nanomedicine on breast cancer bone metastatic. Our initial studies focused on the targeting efficiency of GP_NP_@M to the metastatic bone lesions. Remarkably, a pronounced accumulation of GP_NP_@M at the metastatic site was observed within 2 h of administration, which persisted for up to 24 h (**Figure** **8**A–C). This highlighted its adeptness at bone tumor localization, a cornerstone for successful therapeutic intervention. In terms of therapeutic outcomes, GP_NP_@M significantly impeded tumor expansion in the 4T1‐engrafted limb (Figure 8D–F; Figure S13A, Supporting Information). Complementing these findings, extensive induction of apoptosis was evidenced by the heightened number of TUNEL‐positive cells in tumor sections derived from the GP_NP_@M‐treated ensemble (Figure S13B, Supporting Information). Moreover, 3D reconstructions of tibial structures were generated using micro‐CT to assess osteolytic changes following therapeutic intervention (Figure 8G,H). Subsequent quantitative analyses, specifically evaluating percent bone volume (BV/TV), trabecular thickness (Tb. Th), trabecular number (Tb. N), trabecular separation (Tb. Sp), and bone mineral density (BMD), demonstrated the enhanced efficacy of GP_NP_@M in mitigating osteolytic degradation (Figure 8I). The integrity of the tibial architecture was notably conserved in the GP_NP_@M treatment group, illustrating the protective effect of the nanomedicine against osteolytic assault. In terms of the metabolic regulatory effects, multiplex immunofluorescence staining showed that after GP_NP_@M treatment, the expression of glucose metabolic enzymes in tumors significantly decreased (Figure S14, Supporting Information), which strongly confirmed the metabolic regulatory effect of GP_NP_@M on breast cancer bone metastases.

Based on recent findings showing that the bone microenvironment can exacerbate breast cancer metastasis and facilitate the genesis of secondary metastases in multiple organs,^[^ ^7^ ^]^ we proceeded to quantify the extent of secondary metastatic lesions within the liver and lungs through histopathological staining. Post‐administration of GP_NP_@M, a discernible reduction in tumor metastasis was observed in both liver (Figure 8J) and lung (Figure 8K) tissues of treated mice. Quantitatively, the incidence of liver metastatic nodules in the PBS and GP groups was found to be 3.4‐fold and 2.8‐fold higher, respectively, in comparison to the GP_NP_@M‐treated group (Figure 8L). Also, negligible lung metastatic nodules were counted in the GP_NP_@M treatment group (Figure 8M), highlighting the potential of GP_NP_@M as an effective agent in curtailing distal tumor dissemination.

## 3 Conclusion

We explored that breast cancer establishes a hyperglycemic microenvironment in the bone pre‐metastatic niche before migrating to bone tissue, subsequently intensifying glucose metabolism upon metastatic colonization. We then constructed a biomimetic‐engineered enzyme‐based nanoplatform GP_NP_@M, with the capability of self‐amplifying starvation combined with selective chemotherapy. This platform holds promise for precisely mitigating breast cancer bone metastases and halting distal tumor dissemination. However, the prediction of tumor metastasis sites remains challenging in clinical practice. While this study proposed PET/CT technology as a potential method for predicting bone metastasis sites, direct evidence linking radionuclide signals to metastasis was not thoroughly investigated.

Clinically, the use of combined medications is a common practice and often yields effective results due to synergistic effects. This strategy could be further refined by constructing novel functionalized drug modules. Our approach strategically incorporates PtNP within the internal cavity of GOx (GP), maintaining the enzyme's activity while ensuring an exact 1:1 stoichiometric ratio. Upon internalization by breast cancer cells, GP targets the reduction of intracellular glucose levels, thereby inducing starvation therapy effects. Furthermore, PtNP encapsulated within GOx not only boosts the catalytic activity of GOx but also creates a synergistic effect for selective chemotherapy within the hyperglycemic and inflammatory tumor microenvironment. We subsequently developed a biomimetic nanoplatform based on the engineered enzyme (GP_NP_@M). The membrane shielding of this platform minimizes interactions between GP and circulating glucose.

Overall, this study provides unique insights into the functional role of glucose as a key component of the premetastatic niche and offers a proof‐of‐concept for metabolism‐targeting strategies with extensive implications for precisely mitigating breast cancer bone metastases and halting distal tumor dissemination.

## 4 Experimental Section

### Materials

Tannic acid (TA), Chloroplatinic acid hexahydrate (H_2_PtCl_6_·6H_2_O), PEG (*Mw* = 2000), 3,3′,5,5′‐Tetramethylbenzidine (TMB), Peroxidase from horseradish (HRP), and 4, ’6‐Diamidino‐2‐phenylindole (DAPI) were purchased from Macklin (shanghai, China). Sodium borohydride (NaBH_4_), Glucose, H_2_O_2_ (AR, 30 wt.%), Iron (III) chloride hexahydrate (FeCl_3_·6H_2_O), Tris(4,7‐diphenyl‐1,10‐phenanthroline) ruthenium (II) dichloride complex (Ru(dpp)_3_Cl_2_), and fluorescein isothiocyanate (FITC, 95%) were acquired from Aladdin (Shanghai, China). GOx was obtained from Yuanye Biotechnology (Shanghai, China). Red fluorescent cell membrane probe (Dil), Lyso‐tracker green, Actin‐Tracker Green, Thiazolyl blue tetrazolium bromide (MTT), Mitochondrial membrane potential assay kit (JC‐1), ATP assay kit, Bradford protein assay kit, BCA protein kit and LDH cytotoxicity assay kit were received from Beyotime Biotechnology (Shanghai, China). Calcein/PI cell viability/cytotoxicity assay kit, Nuclear, and cytoplasmic protein extraction kit, and Annexin V‐FITC apoptosis detection kit were purchased from Meilun Biotech (Dalian, China). The glucose kit (hexokinase method) and Lactic Acid assay kit were obtained from Nanjing Jiancheng Bioengineering Institute (Nanjing, China). Detailed antibody information was displayed in the Supplementary table.

### Construction of Breast Cancer Model with Primary and Secondary Bone Metastasis

Balb/c female mice (6‐week‐old) were bought from the Shanghai Model Organisms Center. To establish the orthotopic breast cancer model, 2 × 10^6^ 4T1 cells were inoculated into the right mammary fat pad of the mice. After 5 days of tumor growth, the mice were randomly divided into two groups (*n* = 5). To simulate limb metastasis, 5 × 10^5^ 4T1 cells were injected into the mice's tibia in one group. Similarly, to simulate spine metastasis, 5 × 10^5^ 4T1 cells were injected into the mice's spine in another group.

### PET‐CT Imaging

The images and quantitative statistics data of human PET/CT were obtained from the Department of Nuclear Medicine, Shanghai General Hospital, Shanghai Jiao Tong University School of Medicine. All patients underwent comprehensive medical evaluations to diagnose breast cancer and the postoperative patients were treated with professional surgical procedures. This study belonged to retrospective and anonymous research and was approved by the Human Ethics Committee of Shanghai General Hospital, Shanghai Jiao Tong University School of Medicine.

The tumor‐bearing mice were subjected to a 24 h fasting period prior to imaging. Then, each mouse received an intravenous injection of 8.6 MBq ^18^F‐FDG through the tail vein. PET‐CT was applied 60 min after the injection. After anesthesia with 1–3% isoflurane, the mice were placed on the bed of the PET scanner (United Imaging Inc.). The imaging protocol started with a CT scan, followed by a PET scan. The PET images were reconstructed using the OSEM3D algorithm. For results analysis, regions of interest were selected under CT guidance, and the uptake of ^18^F‐FDG was quantified through inveon research workplace 3.0. All data was shown as the ratio of the standard uptake value in the region of interest (SUV_interest_) to the standard uptake value in the muscle (SUV_muscle_)].

### Targeted Metabolomics Analysis

On day 15 after the initial establishment of the model, tumor tissues from different positions were extracted and immediately preserved in dry ice. After thawing and crushing the sample, 0.05 g of the sample was extracted by mixing it with 500 µL of 70% methanol/water. After multiple centrifugations at 4 °C, 200 µL of the supernatant was analyzed by using ultra‐performance liquid chromatography (UPLC, SCIEX) fitted with an ACQUITY UPLC BEH Amide column (SCIEX). And, the flow rate of the line gradient was 0.4 mL min^−1^ at 40 °C. The UPLC was coupled to tandem mass spectrometry (MS/MS, SCIEX), at a positive/negative voltage (5.5 kV/−4.5 kV) and a curtain gas flow rate of 35 psi. Data acquisition was performed using Analyst 1.6.3 software (Sciex), while metabolite quantification was conducted using Multiquant 3.0.3 software (Sciex).

### Genomic Analysis

After different treatments, 4T1 cells were lysed by TRIzol reagent (Invitrogen) to extract RNA. Then, the mRNA libraries were constructed, and different libraries were sequenced in Illumina (US). Gene expression analysis was performed based on the DESeq2. A hypergeometric test was used for Enrichment analysis. GSEA was generated using the clusterprofiler R package. Gene‐concept network analysis was performed by the ComplexHeatmap R package.

### Synthesis of GP

GP was synthesized based on the preliminary work.^[^ ^34^, ^35^ ^]^ Specifically, 5 mg of GOx and 15 mg of H_2_PtCl_6_·6H_2_O were dissolved in 10 mL of phosphate‐buffered saline (PBS) (pH = 7.5), and the mixture was stirred in darkness for 30 min. Then, 0.25 mL of NaBH_4_ (0.5 m) was added into the reaction system, stirring for 2 h. The reaction mixture was subsequently transferred into a dialysis bag (MWCO 3500 Da) and adequately dialyzed against deionized (DI) water. The purified GP solution was preserved at 4 °C. The content of GOx was estimated by the Bradford method.

### The Obtention of Tumor Cell Membrance

To prepare the 4T1 membrane, 4T1 cells were cultured in dishes, washed three times with sterile PBS, scraped with a cell scraper, and subsequently centrifuged at 3000 rpm for 10 min. The resulting precipitate was resuspended in small‐volume PBS containing PMSF. The resuspension was sonicated by Selecta Sonopuls at 50% power for 2 min in an ice bath (on for 2 s, off for 5 s). Following sonication, the solution was centrifuged at 800 rpm for 10 min (4 °C), and the supernatant was collected. This supernatant was further centrifuged at 5000 rpm for 10 min (4 °C), and the resulting supernatant was retained. Subsequently, the supernatant was subjected to a final centrifugation step at 15000 rpm for 60 min (4 °C). The obtained precipitate was resuspended in a 0.2 mm EDTA aqueous solution. The quantification of membrane proteins was performed using a BCA assay kit.

### Synthesis of GP_NP_ Cores and GP_NP_@M

To prepare GP_NP_, PEG (80 µL, 50 mg mL^−1^), FeCl_3_·6H_2_O (19 µL, 10 mg mL^−1^), and TA (200 µL, 5 mg mL^−1^) were sequentially added to the purified GP solution (2 mL, 0.3 mg mL^−1^) and the mixture was stirred in the room temperature for 30 min. To isolate the GP_NP_, high‐speed centrifugation was employed to remove unbound substances, and the obtained GP_NP_ precipitate was resuspended in deionized (DI) water for further use.

To prepare 4T1‐M‐wrapped GP_NP_ (GP_NP_@M), 0.3 mg GP_NP_ and 0.3 mg 4T1 membrane were dispersed in 1 ml deionized (DI) water. Next, the mixture was extruded 11 times through a 400 nm polycarbonate membrane (Avanti Mini‐Extruder, USA). The quantification of GP, GP_NP_, and GP_NP_@M was performed based on their Pt content.

### Characterization

The ordinary morphology and physical dimension were observed using transmission electron microscopy (TEM, Tecnai G2 spirit Biotwin, USA). The high‐resolution morphology and elemental mapping were detected by field‐emission scanning electron microscopy (FESEM, FEI Talos F200X, USA). The hydrated particle size, zeta potential, and stability of particles in different media (H_2_O, FFM (FBS‐free medium), and FM (FBS‐containing medium)) were measured by dynamic light scattering (DLS, Linkoptik, China). The circular dichroism spectra of protein were obtained with a J‐815 CD spectrometer (Jasco International Co., Japan). The UV–vis spectra were acquired on a UV spectrophotometer (PERSEE, China). The elemental composition and valence were identified by X‐ray photoelectron spectroscopy (XPS, Thermo Fisher, USA). The content of Pt was quantified using inductively coupled plasma (ICP, PerkinElmer, Singapore). The colocalization of two fluorescence was observed by CLSM (Leica TCS SP8, Germany; Dil: *Ex/Em* = 549 nm/565 nm, FITC: *Ex*/*Em* = 488 nm/525 nm). SDS‐PAGE and Coomassie blue fast staining solution were used to confirm the 4T1‐M was coated around the surface of GP_NP_.

### Multifunctional Catalysis of GP

To explore the catalase‐like activity of PtNP, GP or GOx (1 µg mL^−1^, GOx) was dispersed in separate solutions of Glucose (1 mg mL^−1^), H_2_O_2_ (1 mm), and a combined solution of Glucose (1 mg mL^−1^) +H_2_O_2_ (1 mm) solutions. Changes in dissolved oxygen concentration were monitored at different time points using a dissolved oxygen meter. Furthermore, Ru(dpp)_3_Cl_2_, a fluorescence oxygen probe that was quenched by oxygen molecules, was utilized. The above systems were added into a 24‐well plate, with each well receiving Ru(dpp)_3_Cl_2_ (2 mg mL^−1^, 1 µL). The samples were then incubated at 37 °C for 30 min in the dark. Post‐incubation, the fluorescence intensity of Ru(dpp)_3_Cl_2_ in different treatment groups was detected using a small animal live imaging system (IVIS, PerkinElmer, *Ex*/*Em* = 455 nm/615 nm).

To estimate the catalytic effect of GOx on glucose. GP, GOx, GP_NP_, and GP_NP_@M (each with an equivalent GOx concentration of 1 µg mL^−1^) were added to glucose solutions (1 mg mL^−1^) and incubated at 37 °C. The consumption of glucose was determined using a commercial glucose kit. The generation of H_2_O_2_ was measured by colorimetric detection using TMB. Briefly, at specific time points, reaction solution (20 µL) of GP or GOx was collected and mixed with HRP (1 U mL^−1^, 30 µL), TMB (2 mm, 30 µL), and PBS (pH = 5, 150 µL) in a 96‐well plate. This mixture was incubated for 10 min. The absorbance at 652 nm wavelengths was recorded to calculate the H_2_O_2_ concentration. Furthermore, GP (1 µg mL^−1^, GOx) was added into PBS with or without glucose (1 mg mL^−1^). The pH changes in these solutions were continuously monitored using a pH meter.

### Cellular Uptake

Cellular uptake of different treatments was performed using ICP‐MS (Thermo Fisher, USA). GP, GP_NP_, and GP_NP_@M, each containing the equivalent GOx concentration of 0.36 µg mL^−1^ (quantitative determination of GOx based on the content of Pt), were added to cells. After 6 h incubation, 4T1 cells were counted and centrifugation, and the precipitation was utilized for the quantification of Fe and Pt contents using ICP‐MS.

Subsequently, the intracellular accumulation of GP_NP_@M at different time points was analyzed using a flow cytometer (BD Fortessa, USA). Initially, cell membranes were labeled with Dil. Then, cells were treated with GP_NP_@M at the GOx concentration of 0.36 µg mL^−1^. Samples were collected at 2, 4, 6, and 8 h post‐treatment for flow cytometric analysis.

Ultimately, confocal laser scanning microscopy was used to visualize the uptake of GP_NP_@M and its colocalization with endosomes at different time points. 4T1 cells were seeded into confocal dishes and incubated overnight. The treatment with GP_NP_@M followed the same protocol as previously described. After 2, 4, and 6 h treatment, cells were stained with Lysotracker‐green (75 nm) at 37 °C for 30 min. Then, Hoechst (10 µg mL^−1^) was used to label the nuclei before CLSM observation.

Confocal laser scanning microscopy also was used to monitor the uptake of GP_NP_@M in HUVEC, MC3T3‐E1, and RAW264.7 at 4 h. Cells were seeded into confocal dishes and incubated overnight. The treatment with GP_NP_@M followed the same protocol as previously described. After 4 h treatment, cells were stained with Actin‐Tracker Green according to the protocol. DAPI (10 µg mL^−1^) was performed to stain the nuclei.

### Measurement of Metabolic Indicators Content

The original medium was removed, and the cells were subsequently incubated with fresh medium containing GP, GP_NP_, and GP_NP_@M, each at an identical GOx concentration of 0.36 µg mL^−1^, for an additional 24 h. Afterward, the supernatant and cells were harvested separately. The extracellular glucose concentration was tested by a glucose kit, while the intracellular ATP levels were measured with an ATP assay kit.

### Lactic Acid Assay

After 24 h exposure of cells to various treatments, the cells were collected and the intracellular lactate concentration was determined using a lactic acid assay kit, according to the manufacturer's protocols.

### Mitochondrial Membrane Potential Assay

4T1 cells were seeded in confocal dishes and incubated overnight. Subsequent to adding different samples to 4T1 cells for 6 h, changes in the mitochondrial membrane potential were assessed using a mitochondrial membrane potential assay kit (JC‐1). The cells were then imaged using CLSM (JC‐1A: *Ex*/*Em* = 514 nm/529 nm; JC‐1M: *Ex*/*Em* = 585 nm/590 nm) and the relative fluorescence density ratio (%) was calculated by Image J software.

### Seahorse Experiment

The cells were seeded in a seahorse XF cell culture microplate and incubated until reaching the desired cell density. Subsequently, the microplate was detected using a Seahorse XFe Analyzer (Agilent, USA) to measure the OCR and ECAR. The experimental process strictly followed the user guide provided by Agilent.

### Cytotoxicity Assay

After 24 h of exposure to different treatments, cell viability was determined through thiazolyl blue tetrazolium bromide (MTT) assay. Briefly, the supernatant was replaced with the fresh 100 µL MTT‐containing (0.5 mg mL^−1^) culture medium and incubated at 37 °C for 4 h. Subsequently, 100 µL DMSO was added to each well, and the plate was placed on a 96‐well microplate mixer (SCILOGEX, USA) to vibrate thoroughly for 20 min. The spectrometer (Thermo Scientific Varioskan Flash, USA) was used to measure the absorbance at 490 nm.

For the live/dead staining experiment, the cells were stained with Calcein‐AM (2 µm) and PI (8 µm) for 30 min at 37 °C (*n* = 3) and imaged by an inverted fluorescence microscope (Leica, Germany).

For cell apoptosis analysis, the cells were stained with Annexin V‐FITC and PI for 15 min. The flow cytometer was performed to evaluate the intensity of the fluorescence signal.

### Cell Pyroptosis

To investigate the induction of pyroptosis in 4T1 cells, the cells were treated with these materials for 12 h. Morphological changes were documented using bright field imaging on an inverted fluorescence microscope. Additionally, the LDH content in the supernatant was quantified using an LDH cytotoxicity assay kit.

### Western Blotting

Cells and tumor tissues were lysed by RIPA buffer, and protein concentrations were measured using a BCA kit. Then, the loading buffer was added, and the samples were denatured at 100 °C for 10 min. Proteins were then separated by SDS‐PAGE and transferred onto PVDF membranes. The membranes were blocked with 5% skim milk at room temperature for 2 h. The membranes were incubated with the appropriate primary antibodies overnight at 4 °C, then with the second antibodies at room temperature for 2 h. Protein bands were visualized by a chemiluminescence detection system (Tanon 5200, China).

### Immunofluorescence

The cells were fixed with 4% paraformaldehyde for 15 min and permeabilized with 0.5% Triton X‐100 for 30 min at room temperature. The paraffin‐embedded tumor tissues were dewaxed by xylene and rehydrated through a graded alcohol series. Then, both cells and slices were blocked with 5% goat serum for 30 min, followed by overnight incubation with primary antibodies. Next, the fluorescently labeled secondary antibodies were used, and the samples were stained with DAPI (10 µg mL^−1^) for 10 min. CLSM was applied to capture the fluorescent images.

### Detection of Intranuclear Pt and Pt^II^

4T1 cells were seeded in 10 cm dishes and incubated in three different media conditions: high‐glucose medium (4.5 g L^−1^) with or without H_2_O_2_ (100 µm), and low‐glucose medium (1 g L^−1^), each containing GP_NP_@M (with a GOx content of 0.36 µg mL^−1^) for 6 h. After collecting and counting the cells, the cytoplasm and nucleus were isolated using a nuclear and cytoplasmic protein extraction kit. ICP‐MS was applied to detect the content of intranuclear Pt. Additionally, GP was dispersed in a glucose‐H_2_O_2_ solution and incubated at 37 °C for 24 h before analyzing the valence of Pt by XPS.

### Cell Colony Assay

4T1 cells were treated with H_2_O_2_ (100 µm), GP_NP_@M (with a corresponding GOx concentration of 0.36 µg mL^−1^), and GP_NP_@M +H_2_O_2_ for 24 h. After digesting and counting, 1200 cells per well were seeded and cultured for 7 days. Colonies were stained with crystal violet and counted by image J.

### Cell Cycle Analysis

The processed 4T1 cells were fixed in ice‐cold 75% ethanol overnight. Subsequently, the cells were treated with RNase A and PI for 30 min at 37 °C and then analyzed by flow cytometer.

### Anti‐Tumor Efficacy In Vivo

For the orthotopic tumor model, 2 × 10^6^ 4T1 cells were suspended in 100 µL PBS and subcutaneously injected into the right dorsal flank of each mouse. When the tumor volume reached ≈50–80 mm^3^, mice were randomly divided into four groups and administrated with PBS, GP, GP_NP_, and GP_NP_@M (at the same GOx concentration was 22.5 µg mL^−1^, 200 µL) via intravenous injection. The frequency of treatments was once every two days, with a total of 5 treatments. The tumor volume and body weight of the mice were recorded during the experimental period. On day 18, the mice were sacrificed, and tumors were excised, weighed, and photographed.

For the bone metastasis model, 5 × 10^5^ 4T1‐Luc cells were suspended in 20 µL PBS and injected in situ into the bone marrow cavity of the tibia. Five days after injection, the mice were administered intraperitoneally with 3 mg *D*‐luciferin (PerkinElmer) and monitored by using IVIS, respectively. The mice with similar luminescence intensity were applied to the next therapeutic experiments. For therapeutic analysis, the mice were randomly divided into three groups and administrated with PBS, GP, and GP_NP_@M (at the same GOx concentration of 22.5 µg mL^−1^, 200 µL) via intravenous injection. The frequency of treatments was also once every two days, with a total of 5 treatments. The circumference of the tumor‐bearing leg was measured after each administration. On day 18, the mice were sacrificed, and the tumor‐bearing legs were excised, weighed, and photographed.

### Immunohistochemistry

The paraffin‐embedded tumor tissues were dewaxed by xylene and rehydration in alcohol. The tumor slices were then incubated with primary antibody and subjected to chromogenic detection by DAB. Next, cell nuclei were counterstained with hematoxylin.

### Biodistribution of GP_NP_@M

Three bone tumor‐bearing mice were intravenously injected with Cy5 labeled GP_NP_@M. The fluorescence signal was detected by IVIS at 2, 4, 8, 12, and 24 h, respectively. After 24 h, the main tissues including the heart, liver, spleen, lung, kidney, and tumor‐bearing leg were obtained and also imaged by using IVIS.

### Micro‐CT

Micro‐CT imaging (Bruker skyscan1176, USA) was conducted to evaluate the degree of bone destruction after different treatments. Meanwhile, quantitative statistics of specific bone parameters, such as BV/TV, Tb.Th, Tb.N, Tb.Sp, and BMD, were performed.

### Secondary Metastatic Studies

After therapeutic treatment, the livers and lungs of bone tumor‐bearing mice were used for HE staining, and the secondary metastatic lesions were counted by C.V2.4.

### Biosafety Evaluation

The healthy mice were randomly divided into two groups and treated with PBS or GP_NP_@M, using the same dosage as before. Treatments were administered once every two days for a total of 5 times. After 18 days, the whole blood samples of the mice were collected for routine blood tests, and the serum samples were taken for biochemical tests. Additionally, the major organs including hearts, livers, spleens, lungs, and kidneys were harvested for HE staining.

Hemolytic activity was assessed by incubating freshly isolated mouse erythrocytes with GP_NP_@M nanoparticles (GOx concentration 0.67–10 µg mL^−1^) in saline at 37 °C for 1 h. Following centrifugation (2000× rpm, 5 min), the EP tubes were photographed and the hemoglobin release was quantified through spectrophotometric measurement at 540 nm, with 1% Triton X‐100 and saline serving as positive and negative controls, respectively.

### Statistical Analysis

All quantitative analyses were conducted with at least three biological replicates. Quantitative data were presented as mean ± s.e.m. Statistical analysis was calculated using unpaired Student *t‐*test, one‐way ANOVA with Tukey's multiple‐comparisons test, and two‐way ANOVA with Šidák's multiple‐comparison by GraphPad Prism 8.0. *P *< 0.05 indicated statistical significance.

## Conflict of Interest

The authors declare no conflict of interest.

## Author Contributions

J.Y., P.H., and R.Z. contributed equally to this work. J.Y., P.H., C.W., and D.S. designed the research. J.Y., P.H., L.Z., Y.C., S.R., Y.Q, and M.Z. performed the experiments. J.Y. wrote the manuscript. J.Y. and R.Z. analyzed the data. X.Y., C.W., and D.S. revised the manuscript. R.Z., Z.L., H.D., and X.Y. conducted PET/CT imaging. T.M. provided the human specimen analysis.

## Supporting information



Supporting Information

## Data Availability

All data supporting the findings of this research are available in the article and Supplementary Information. All data obtained during the research period can be provided from the corresponding authors for research requests.
