# Supplementary material for: Targeting Hyperglycemic Bone Pre‐Metastatic Niche for Breast Cancer Bone Metastasis Therapy
Source: Adv Sci (Weinh). 2025 Jun 10;12(37):e04924. doi: 10.1002/advs.202504924 (PMC12499427; doi:10.1002/advs.202504924)
Supplement: Supplementary file 1 — Supporting Information [file ADVS-12-e04924-s001.docx]

**Supplementary table.** Antibodies information.

| **Antibodies** | **Company** | **Catalog #** | **Application** |
| --- | --- | --- | --- |
| Hexokinase I Rabbit mAb | CST | C35C4 | WB/IF |
| Pyruvate Dehydrogenase Rabbit mAb | CST | C54G1 | WB/IF |
| Phospho-Chk1 (Ser345) (133D3) Rabbit mAb | CST | 2348 | WB |
| Recombinant Anti-DFNA5/GSDME antibody [EPR19859]-Nterminal | Abcam | ab215191 | WB |
| Recombinant Anti-HMGBl antibody [EPR3507] | Abcam | ab79823 | IF |
| Recombinant Anti-NLRP3 antibody [EPR23094-1] | Abcam | ab263899 | WB |
| Recombinant Anti-PARP1 antibody [EPR18461] | Abcam | ab191217 | WB |
| Recombinant Anti-gamma H_2_A.X (phospho S139) antibody [EP854(2)Y] | Abcam | ab81299 | WB/IF |
| Anti-Ki67 antibody | Abcam | ab15580 | IHC |
| Transketolase Polyclonal antibody | Proteintech | 11039-1-AP | WB/IF |
| ASL Polyclonal antibody | Proteintech | 16645-1-AP | WB/IF |
| Hexokinase II Polyclonal antibody | Proteintech | 22029-1-AP | WB |
| Beta Actin Monoclonal antibody | Proteintech | 66009-1-Ig | WB |
| CALR Antibody | Affinity | DF3139 | WB/IF |
| Cleaved-Caspase3 (Asp175), p17 Antibody | Affinity | AF7022 | WB/IHC |
| HIF1A Antibody | Affinity | AF1009 | WB/IF |
| Tubulin beta Antibody | Affinity | AF7011 | WB |


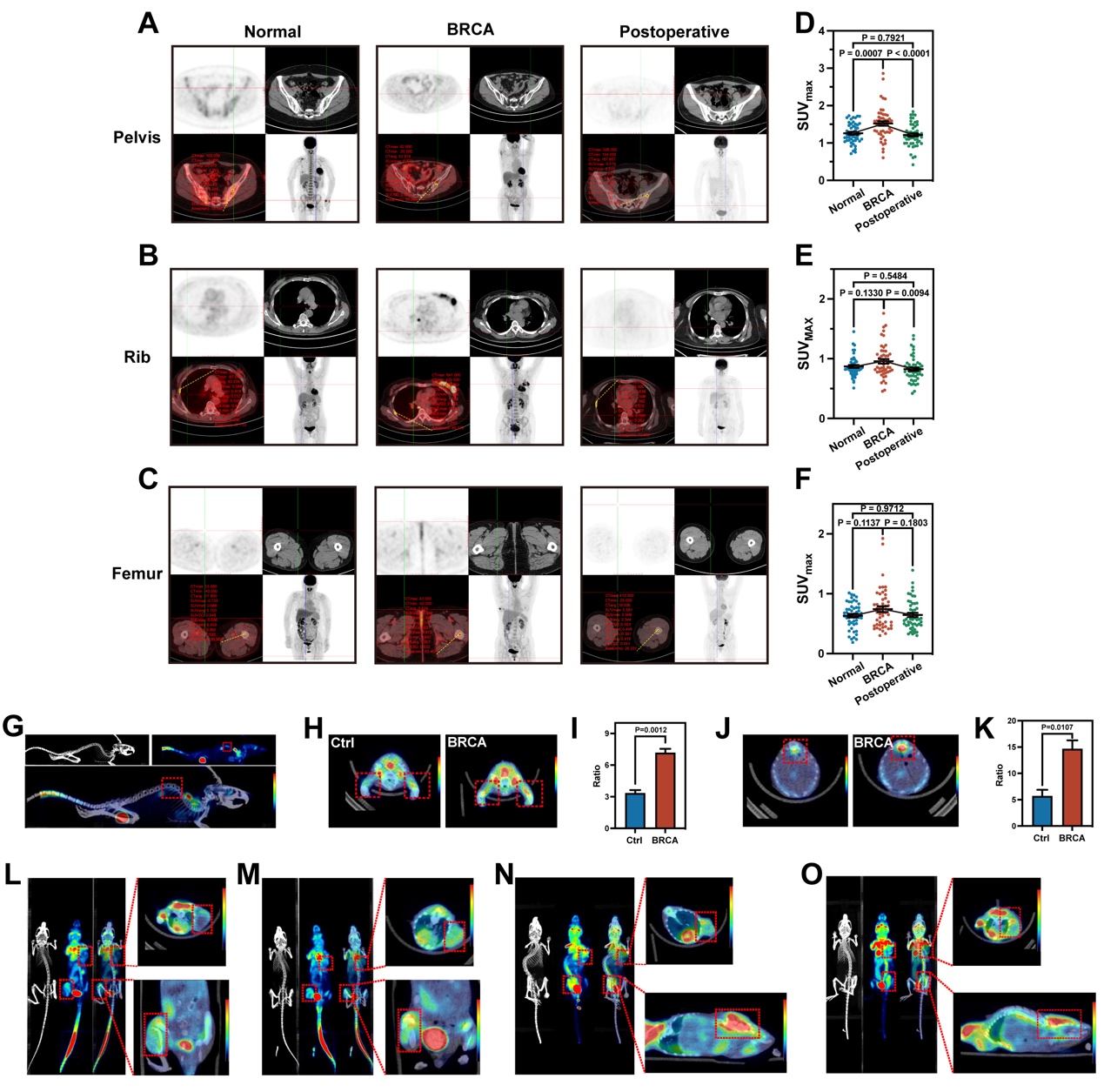


**Figure S1. ^18^F-FDG uptake of the breast cancer patients and tumor mice.** A-C) The ^18^F-FDG PET-CT images of the pelvis (A), rib (B), and femur (C) in healthy individuals, breast cancer patients, and postoperative breast cancer patients. D-F) Corresponding^18^F-FDG uptake quantification (n=50 independent samples, mean ± s.e.m.). G) Maximum intensity projection (MIP) of the ^18^F-FDG PET in normal mice. H, I) Axial FET/CT images (H) and ^18^F-FDG uptake quantification (I) in the forelimb of normal mice and BRCA mice (n=3 independent samples, mean ± s.e.m.). J, K) Axial PET/CT images (J) and ^18^F-FDG uptake quantification (K) in the thoracic vertebrae of normal mice and BRCA mice (n=3 independent samples, mean ± s.e.m.). L, M) PET/CT imaging of two additional mice from Figure 2C. N, O) PET/CT imaging of two additional mice from Figure. 2E. P values were calculated by one-way ANOVA with Tukey’s multiple-comparisons test (D-F) and two-tailed unpaired Student t-test (I, K)


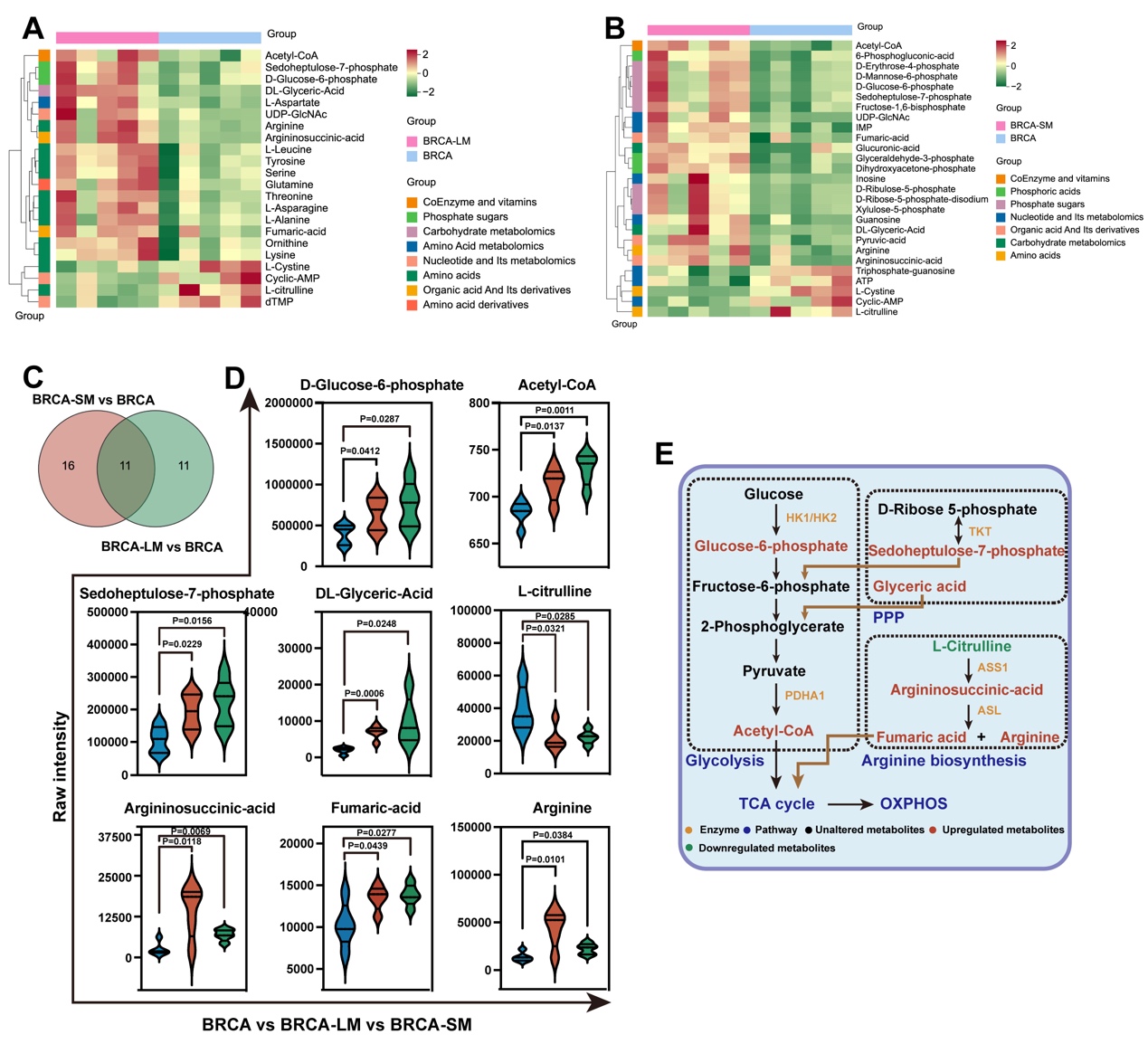


**Figure S2. Metabolomics analysis of primary and bone metastasis breast cancer.** A, B) Heatmaps of differential metabolites between two comparative groups, BRCA-LM vs BRCA (A); BRCA-SM vs BRCA (B). C) Venn diagram of differential metabolites in two comparison groups. D) Violin diagram of metabolites closely related to energy metabolism in common differential metabolites (n=5 independent samples). E) Schematic representation of pathways of common differential metabolites and key enzymes. P values were calculated by two-tailed unpaired Student t-test (D)


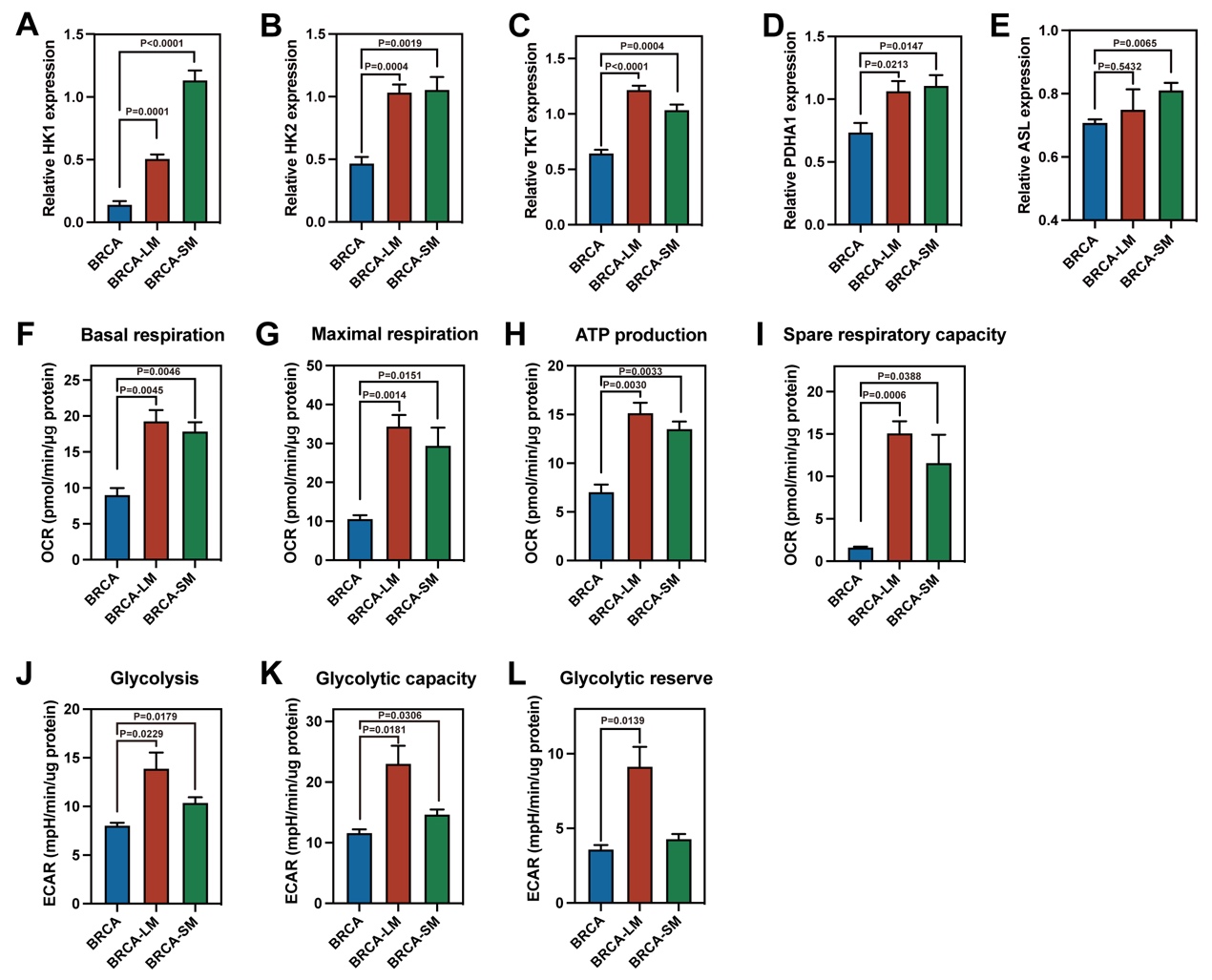


**Figure S3.** A-E) Quantitative statistics of Figure 2M (n=4 independent samples, mean ± s.e.m.). F-L) Parameter detection of OCR in Figure 2N (F-I) and ECAR in Figure 2O (J-L) (n=3 independent samples, mean ± s.e.m.). P values were calculated by two-tailed unpaired Student t-test (A-L).


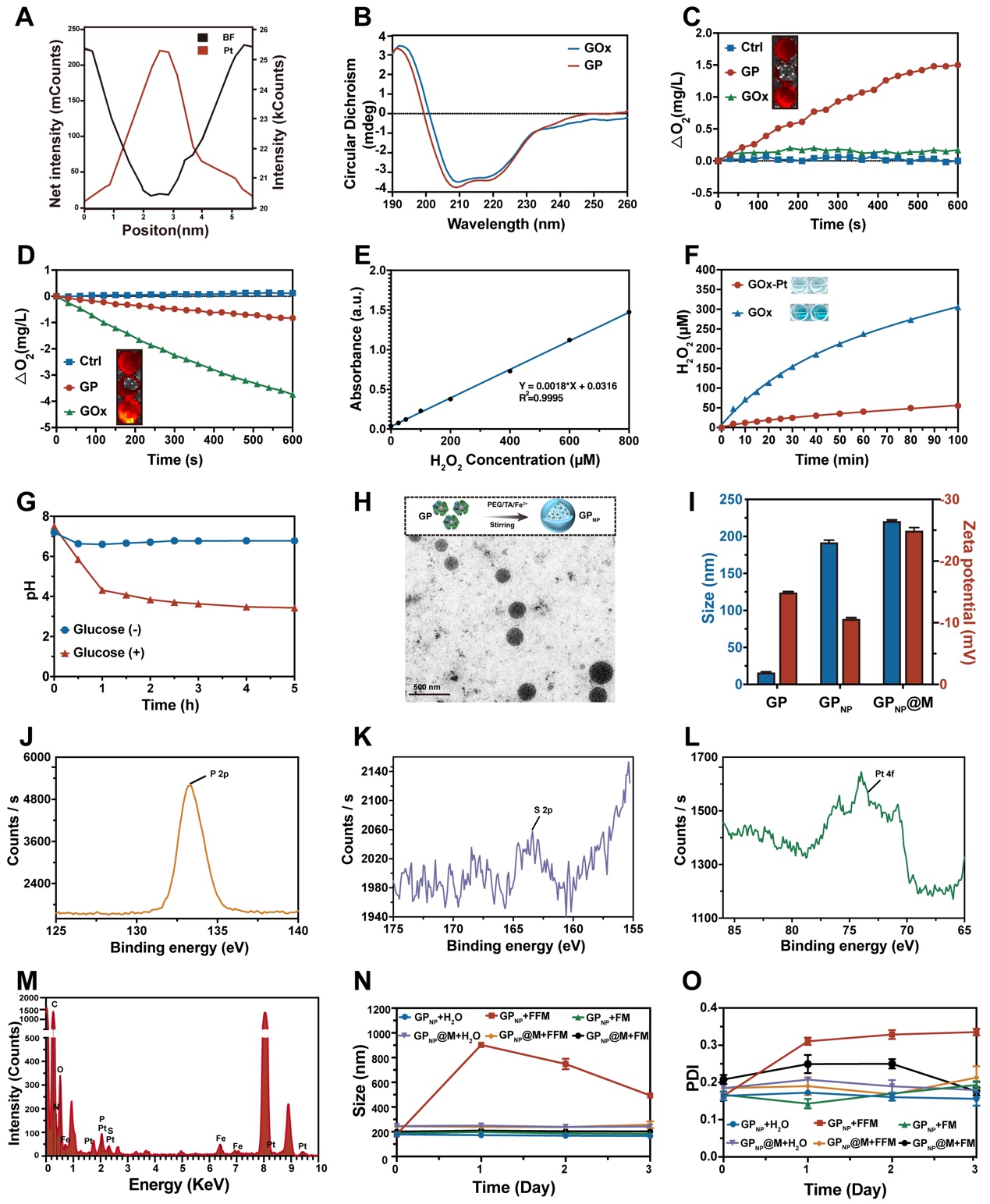


**Figure S4. Characterization of GP and GP_NP_@M.** A) Element line-scan analysis (yellow arrow in Figure 2B) by EDS spectrometry. B) Circular dichroism spectrum of GP and GOx. C, D) Oxygen generation by GP and GOx in a single H_2_O_2_ solution (C) and a single glucose solution (D), with IVIS images of the oxygen probe in different conditions displayed in the inset. E, F) Production of H_2_O_2_ by GP and GOx in glucose solution. G) pH changes in GP solution with and without glucose. H) TEM image of GP_NP_. I) Size and zeta potential of GP, GP_NP_, and GP_NP_@M (n=3 independent samples, mean ± s.e.m.). J-L) Narrow-scan spectrum of P 2p (J), S 2p (K), and Pt 4f (L) in GP_NP_@M. M) EDS element analysis of GP_NP_@M. N, O) Changes in size (N) and PDI (O) of GP_NP_ and GP_NP_@M in different media (n=3 independent samples, mean ± s.e.m.).


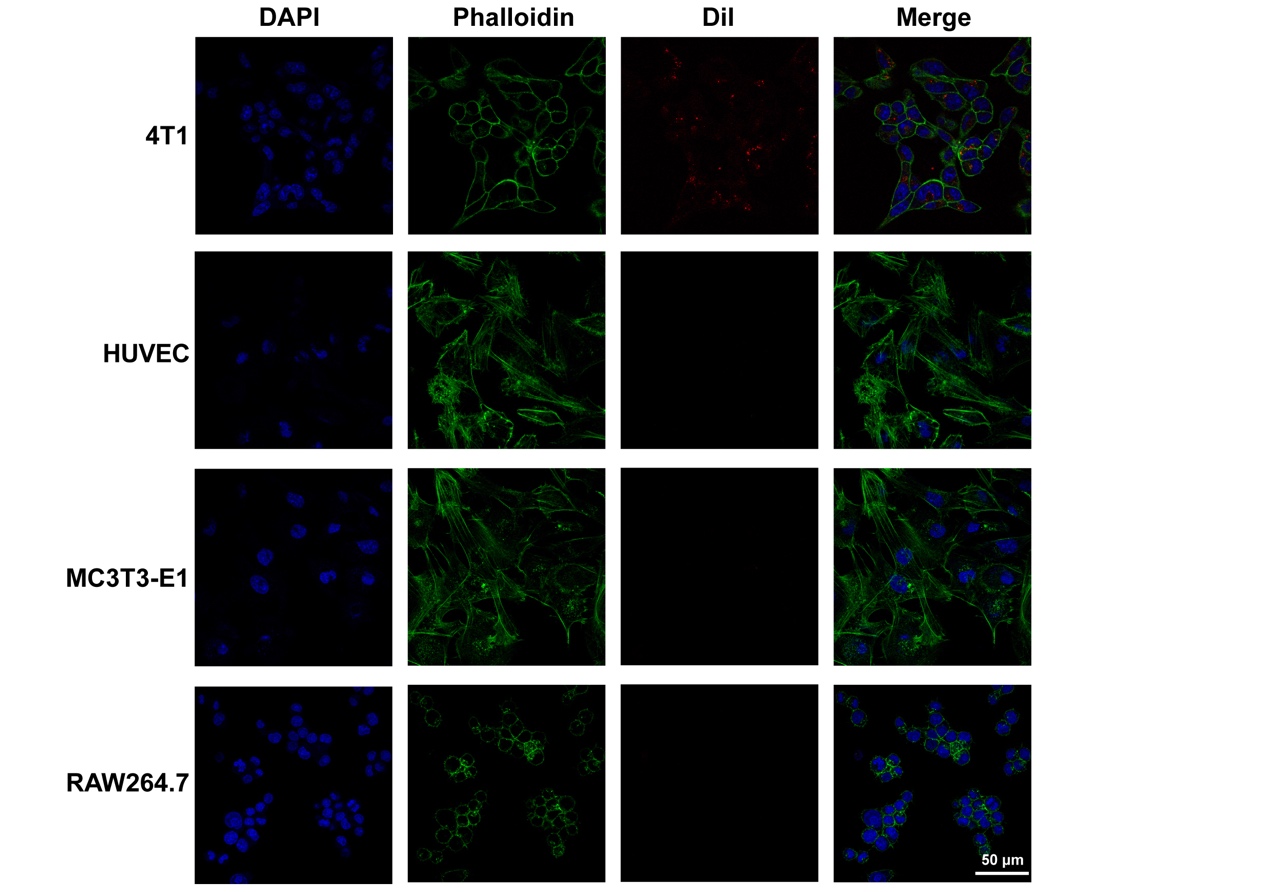


**Figure S5.** Internalization of GP_NP_@M in different cells. (Green: Actin; Red: Dil-labeled GP_NP_@M).


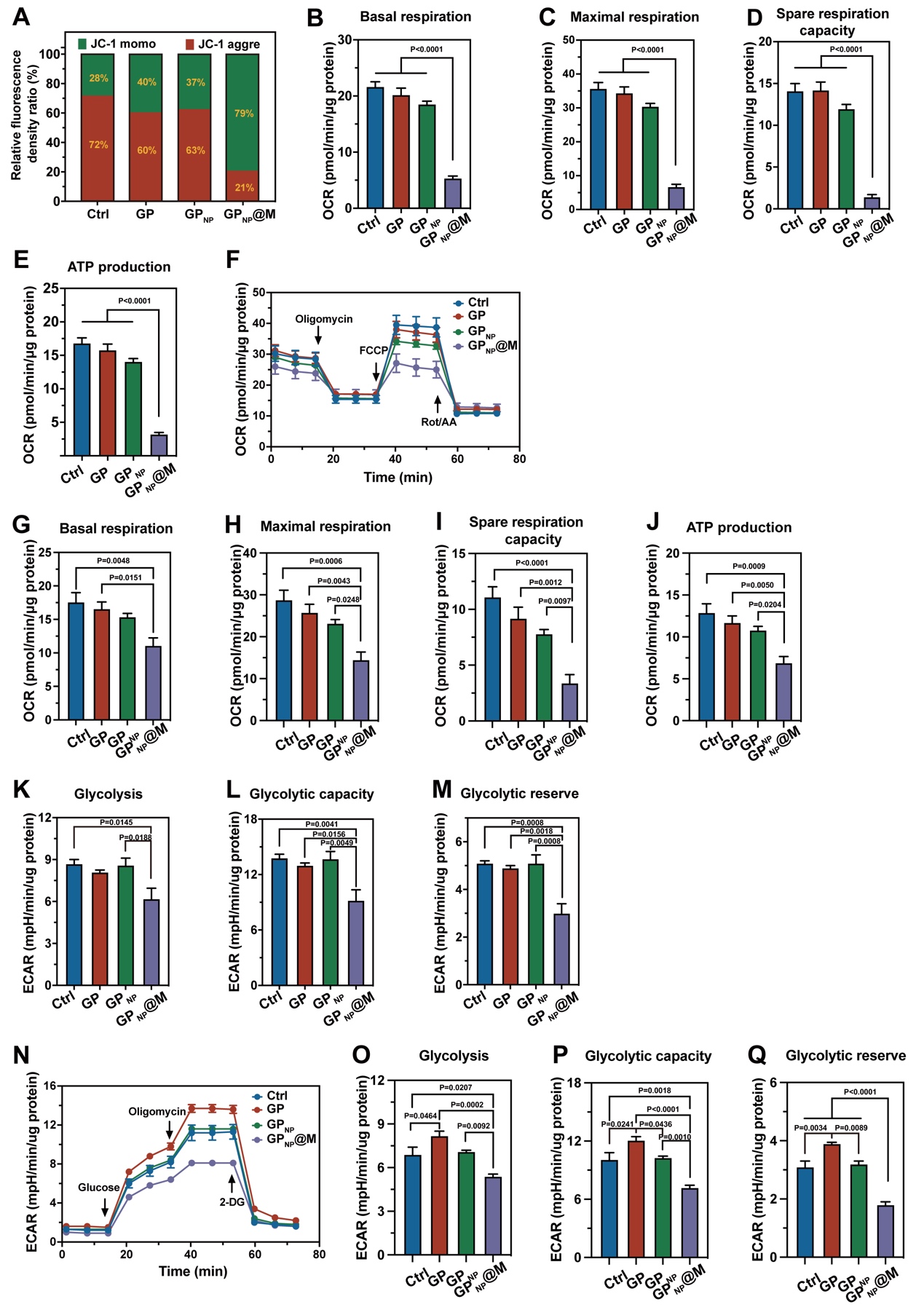


**Figure S6. Starvation effect of GP_NP_@M.** A) Quantitative analysis of JC-1 staining in Figure 4D. B-E) Parameter detection of OCR in Figure 4E. F-J) The levels of OCR (F) in 4T1 cells after different treatments for 12h and relative parameter (G-J). K-M) Parameter detection of ECAR in Figure 4F. N-Q), The levels of ECAR (N) in 4T1 cells after different treatments for 12h and relative parameter (O-Q). P values were calculated by one-way ANOVA with Tukey’s multiple-comparisons test (B-E, G-M, and O-Q). Data were shown as the mean ± s.e.m and n=4 biologically independent.


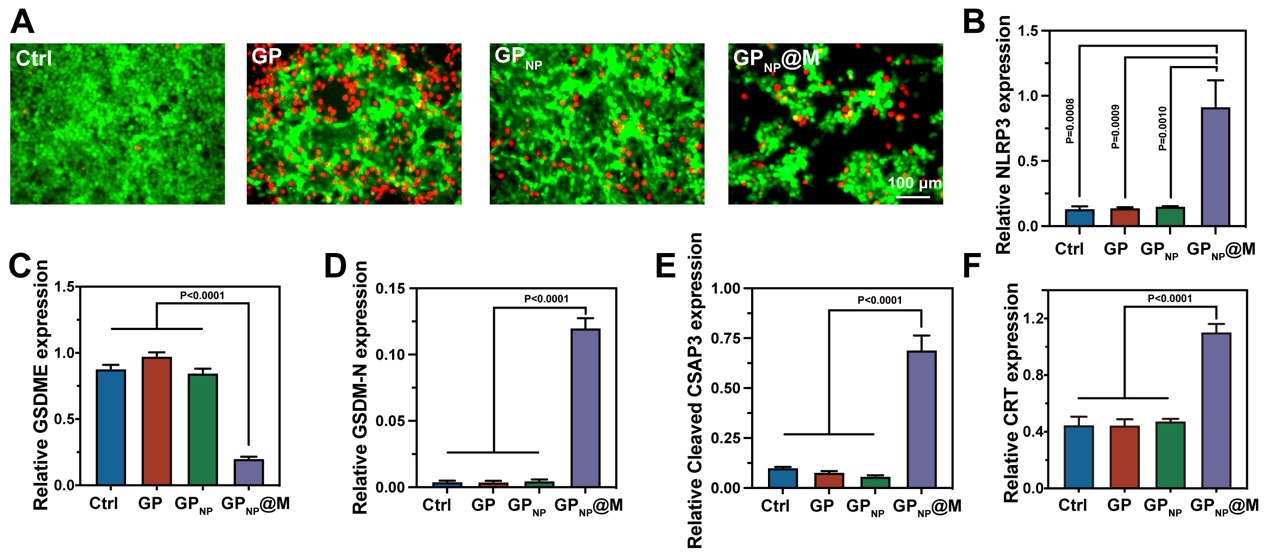


**Figure S7.** **GP_NP_@M induced pyroptosis.** A) Live/dead staining of 4T1 cells after treatment with PBS, GP, GP_NP_, and GP_NP_@M. B-F) Corresponding quantified results of western blotting in Figure 4K. P values were calculated by one-way ANOVA with Tukey’s multiple-comparisons test, data were shown as the mean ± s.e.m, and n=4 biologically independent (B-F). In A, representative results were displayed from at least triplicate independent experiments.


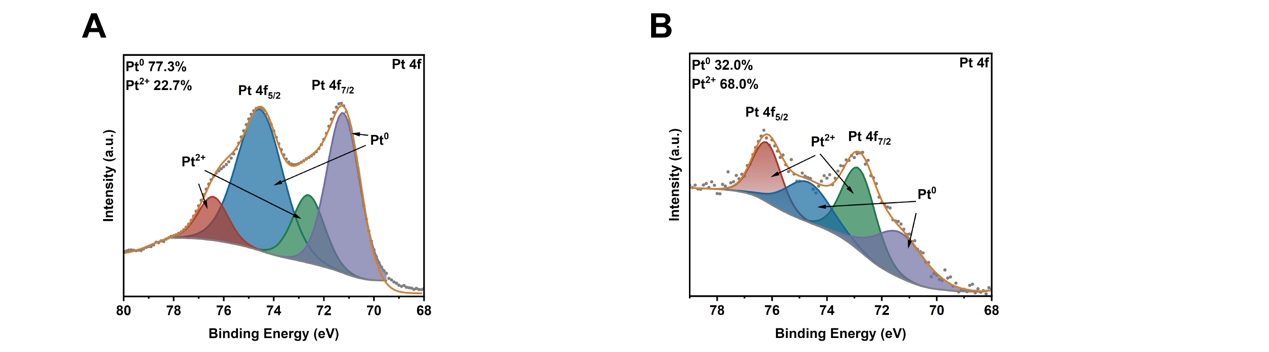


**Figure S8.** A, B) The Pt valence state analysis of GP before (A) and after (B) treatment with glucose plus H_2_O_2_ solution. Representative results were displayed from at least triplicate independent experiments.


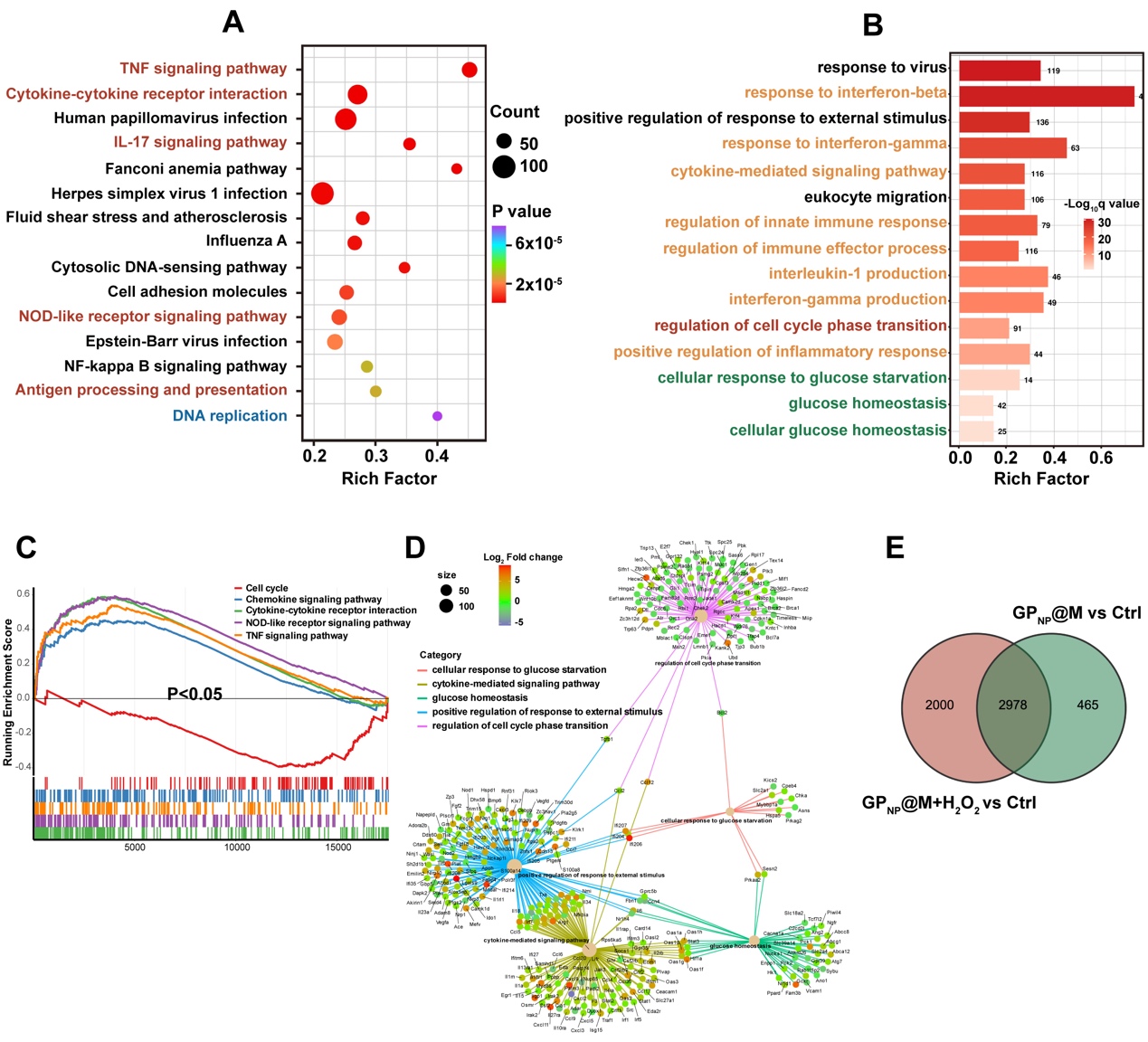


**Figure S9.** A-D, KEGG pathway enrichment analysis (A), GO analysis (B), GSEA analysis (C), and gene-concept network plot (D) of DEGs between the GP_NP_@M treatment group and control group. E) Venn diagram of DEGs in two comparison groups. For each plot, n=3 independent samples.


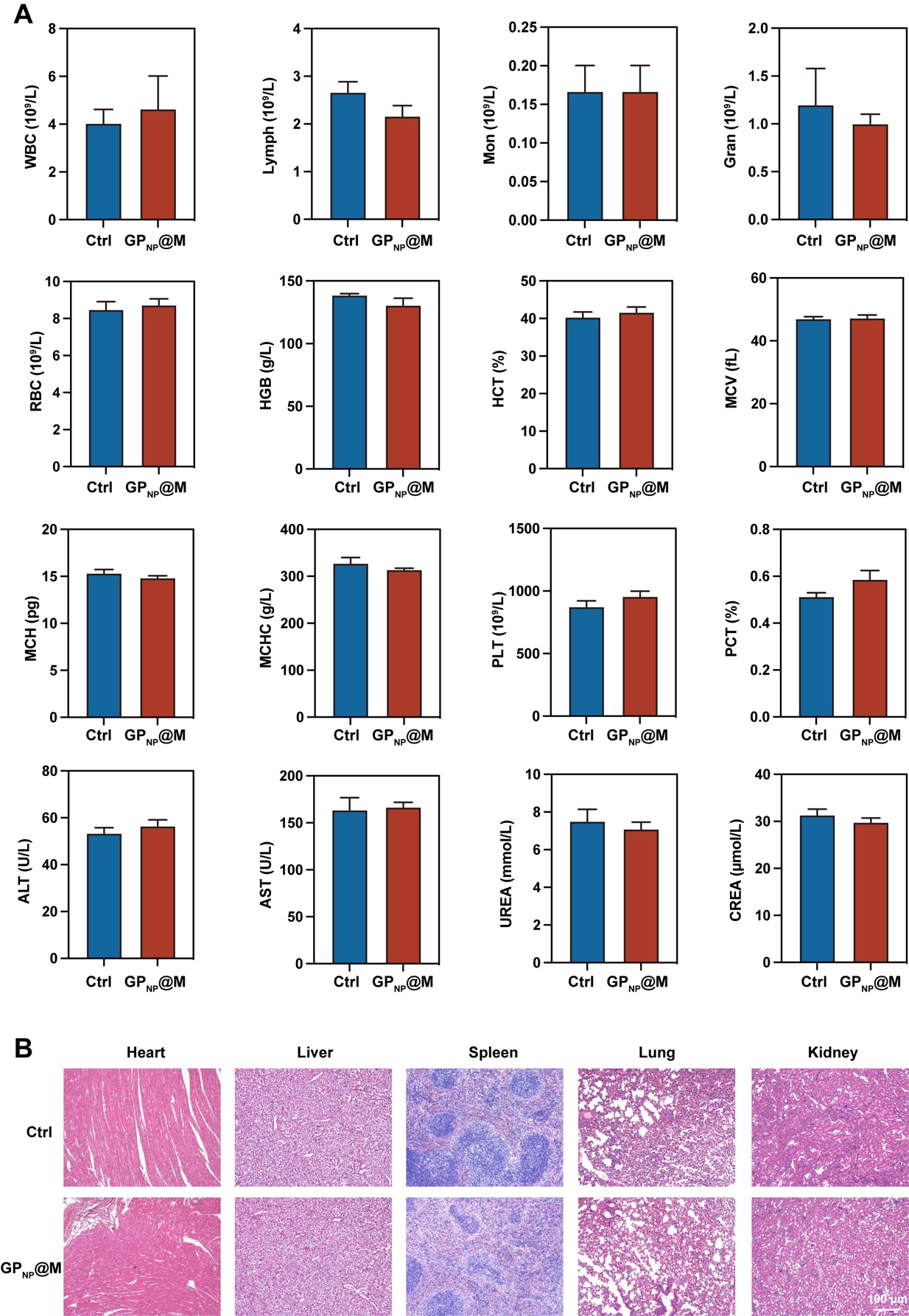


**Figure S10. Biosafety evaluation of GP_NP_@M.** A, B) Assessment of the blood routine and blood biochemistry (A), and the HE staining images of major organs (hearts, livers, spleens, lungs, and kidneys) (B) from the healthy mice injected with PBS and GP_NP_@M. P values were calculated by two-tailed unpaired Student t-test, data were shown as the mean ± s.e.m, and n=3 biologically independent (A). In B, representative results were displayed from at least triplicate independent experiments.


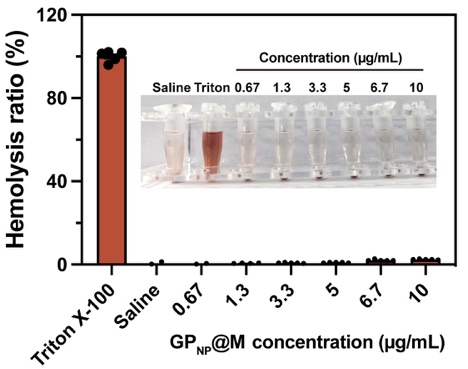


**Figure S11.** The hemolysis analysis of GP_NP_@M at various GO_X_ concentrations.


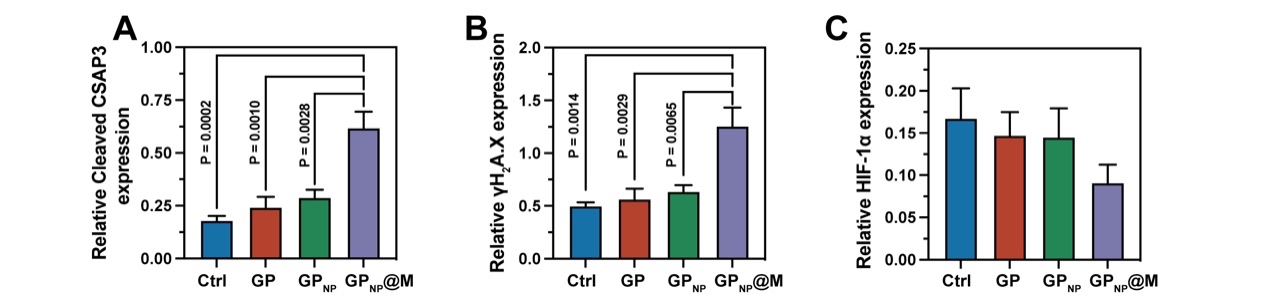


**Figure S12.** A-C) Quantified western blotting results from Figure 7F, J, and M. P values were calculated by one-way ANOVA with Tukey’s multiple-comparisons test. Data were shown as the mean ± s.e.m. n=4 biologically independent.


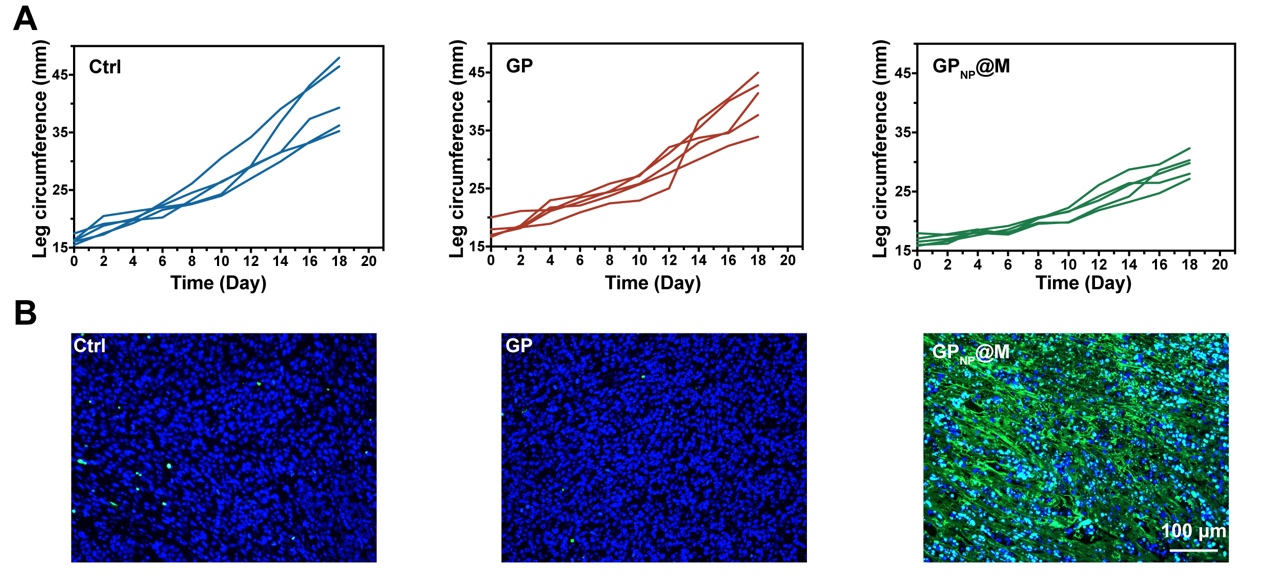


**Figure S13.** A) Circumference changes in tumor-bearing legs of each mouse among three treatment groups in Figure 8D. B) TUNEL staining of tumor slices from the mice with bone tumors after various treatments. In B, representative results were displayed from at least triplicate independent experiments.


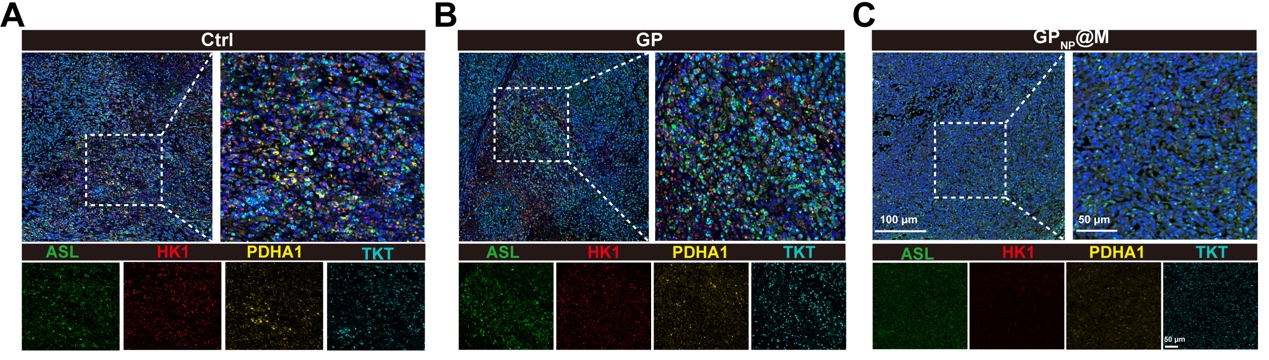


**Figure S14.** The immunofluorescence staining of bone tumors in the mice after various treatments.
